# A Simple yet Efficient Hydrophilic Phenanthroline-Based Ligand for Selective Am(III) Separation under High Acidity

**DOI:** 10.1021/acscentsci.3c00504

**Published:** 2023-07-14

**Authors:** Deshun Tian, Yaoyang Liu, Yu Kang, Yue Zhao, Pengcheng Li, Chao Xu, Li Wang

**Affiliations:** † Department of Chemistry, Capital Normal University, Haidian District, Beijing 100048, People’s Republic of China; ‡ Institute of Materials for Optoelectronics and New Energy, Hubei Key Laboratory of Plasma Chemistry and Advanced Materials, School of Materials Science and Engineering, Wuhan Institute of Technology, Wuhan, Hubei 430205, People’s Republic of China; § Institute of Nuclear and New Energy Technology, Tsinghua University, Haidian District, Beijing 100084, People’s Republic of China; ∥ CAS Key Laboratory of Green Process and Engineering, State Key Laboratory of Biochemical Engineering, Institute of Process Engineering, Chinese Academy of Sciences, Haidian District, Beijing 100190, People’s Republic of China

## Abstract

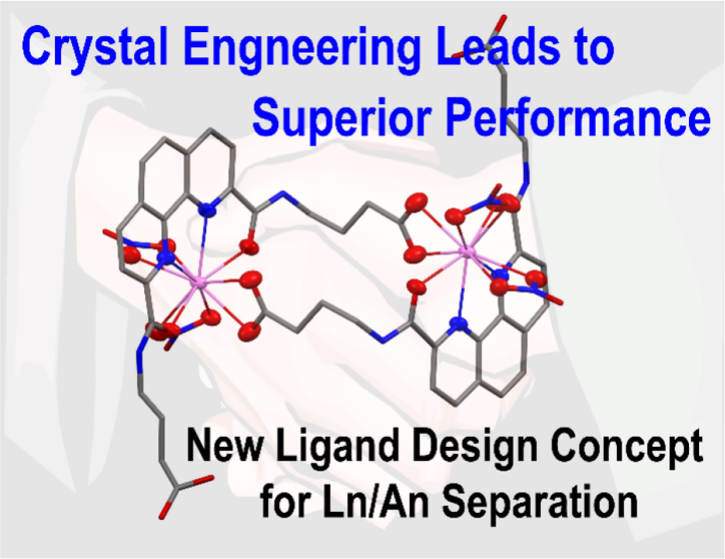

Highly selective hydrophilic ligands were believed to be an efficient way to overcome the massive amount of hazardous organic solvent used in the liquid–liquid extraction process and stood as a new frontier in the Lns(III)/Ans(III) partition. Current reported hydrophilic ligands suffer from harsh preparation conditions, inferior extraction performances, limited available chemical structures, and inability to carry out extraction under high acidity. In this article, we report a simple yet efficient carboxylic group modified phenanthroline-diimide ligand which displayed unexpected Lns(III)/Ans(III) and Ans(III)/Ans(III) separation capabilities in 1.5 M HNO_3_. Unique dimeric architectures for Eu(III) complexes were observed, which could be the origin of the outperforming selectivity and acid resistance. We believe this crystal engineering approach could inspire a renaissance in searching for new functional groups and coordination modes for efficient, high-acid-tolerance Lns(III)/Ans(III) separation ligands.

## Abstract 2

Elaborated ligand design by crystal engineering leads to highly efficient and acid-tolerant hydrophilic Lns(III)/Ans(III) partitioning ligands.

## Introduction

Nuclear power is a vitally important alternative energy source featuring a high power density and less greenhouse-gas emission. However, the operation of nuclear power plants generates enormous amounts of radioactive and heat-releasing spent nuclear fuel (SNF) which should be handled properly for sustainable development and relief of public concerns.^1,2^ The SNF produced from the current popular light water reactors mainly contains uranium (U), plutonium (Pu), and other fission products (lanthanides (Lns), Sr, Cs, etc.) together with less than 1% of the minor actinides (Ans).^2,3^ U and Pu can be mostly removed by the plutonium uranium reduction extraction (PUREX) process, and the remaining raffinate is highly radioactive, contributed mainly by minor amounts of Ans. This high-level liquid waste (HLLW) is then subjected to the partitioning and transmutation (P&T) process to separate the radioactive minor Ans chemically and transmute them into short-lived nuclides though neutron bombarding. The prerequisite for P&T lies in the efficient separation of Lns from Ans due to the large neutron cross section of Lns.^2,4−14^

Partitioning Lns and Ans is one of the most challenging hydrometallurgical separations known due to their chemical/physical similarities.^7,8,10,11,13,15,16^ The high acidity and radioactivity of the HLLW further worsen the situation.^17−19^ Indeed, Lns(III)/Ans(III) separation was once believed to be an impossible mission until Musikas et al. reported their seminal observation on the preferential complexation of Ans(III) over Lns(III) by azide or *ortho*-phenanthroline and on the selective extraction of Am(III) over Eu(III) by 2,4,6-tris(pyridin-2-yl)-1,3,5-triazine (**TPTz**).^4,5^ This opened the gate to the search for multidentate *N*-donor ligands for selective Lns(III)/Ans(III) separation. Representative ligands such as 2,6-bis(5,6-dialkyl-1,2,4-triazin-3-yl) pyridine (**BTPs**),^20,21^ 6,6′-bis(5,5,8,8-tetramethyl-5,6,7,8-tetrahydrobenzo[*e*][1,2,4]triazin-3-yl)-2,2′-bipyridine (**CyMe4-BTBP**),^22,23^ and 2,9-bis(5,5,8,8-tetramethyl-5,6,7,8-tetrahydrobenzo[*e*][1,2,4]triazin-3-yl)-1,10-phenanthroline (**CyMe4-BTPhen**)^24^ were chronologically developed, and great success was achieved. Although these multidentate *N*-donor ligands showed high affinity for Am(III) over Eu(III), they were inferior for light actinides (U, Np, Pu) and, at the same time, the high affinity for Am(III) made the stripping processes very challenging.^18^ Under such circumstances, ligands bearing both soft N and hard O atoms were developed.^6,17,18,25,26^ Represented by the ligand of *N,N′*-diethyl-*N,N′*-ditolyl-2,9-diamide-1,10-phenanthroline (**Et-Tol-DAPhen**), the new tetradentate *N*,*O*-donor ligands displayed efficient Ans(III) extraction abilities and improved selectivity toward group extraction of Ans(III). However, poor solubility in nonpolar solvents (kerosene and *n*-dodecane) impeded these lipophilic ligands for further industrial application.^27^

An alternative Lns(III)/Ans(III) separation strategy was to extract both Lns(III) and Ans(III) unselectively with a diglycolamide such as **TODGA**, followed by selective back-extraction of Ans(III) using a delicate-designed hydrophilic ligands as demonstrated in the Innovative Selective Actinide Extraction (*i*-SANEX) process, trivalent actinide lanthanide separation with phosphorus-reagent extraction from aqueous komplexes (TALSPEAK), process, and group actinides extraction (GANEX) process.^3,28−33^ The sulfonated multidentate *N*-donor ligands (**SO**_**3**_**-Ph-BTP**, **TS-BTPhen2**, and **DS-Ph-DAPhen**, as shown in Scheme S1) were among the most explored hydrophilic ligands.^27,30,34−37^ The separation factors for Eu(III) and Am(III) (SF_Eu/Am_) of these sulfonated ligands could reach as high as 10^3^ in acid and the selectivity was reported to be closely related to the number of sulfonate groups.^34^ Drawbacks of these ligands included harsh reaction conditions for the introduction of sulfonate groups and the secondary waste generated by sulfur, conflicting with the CHON concept. Thus, CHON-compatible ligands bearing hydroxyl groups were developed. Ligands derived from pyridine (**PyTri**),^28^ bipyridine (**EtOH-BPTD**),^38^ and phenanthroline (**BTrzPhen**)^39^ were successively reported. Reasonable SF_Eu/Am_ values of mostly around 50 were demonstrated with satisfying capability of Lns(III)/Ans(III) and intra-actinide (**EtOH-BPTD** and **BTrzPhen**) discrimination. The hydroxyl groups were introduced by a click reaction between ethynyl-functionalized *N*-donor ligands and the corresponding azides, which, chemically, were explosive and not suitable for large industrial production. Furthermore, almost all of the reported hydrophilic ligands were only effective under low acidity, while the raw PUREX raffinate solution was in approximately 3–4 mol/L HNO_3_. The separation factors dropped dramatically with increasing acidity because of the protonation of the ligands and reverse shifts of the coordination equilibrium at high HNO_3_ concentration.^5−7,24^

Inspired by the successful application of diethylenetriaminepentaacetic acid (**DTPA**, structure shown in Scheme S2) in the TALSPEAK process^40^ and the fact that carboxylic groups were frequently used structural motifs in the design of water-soluble Lns complexes,^41−43^ we herein propose, for the first time, a carboxylic group modified hydrophilic phenanthroline-based tetradentate *N*,*O*-ligand which displays extraordinary Lns(III)/Ans(III) and Ans(III)/Ans(III) separation capabilities under high acidity (over 1.5 M HNO_3_). Record high SF_Eu/Am_ values of 120 and SF_Cm/Am_ values of 4.4 were observed in 1.5 M HNO_3_ when the new hydrophilic ligand was used as a masking agent in combination with **TODGA** in dodecane as the organic extracting phase. The coordination behaviors of the new ligand were demonstrated with UV–vis absorption spectroscopy, nuclear magnetic resonance spectroscopy (NMR), and time-resolved laser fluorescence spectroscopy (TRLFS) titrations. Both 1:1 and 1:2 (Eu(III)/ligand ratio) species were detected, which was further confirmed by high-resolution mass spectroscopy (HRMS). Single crystals of both ligand and Eu(III) complexes were solved and the results echoed with our molecular design that the carboxylic group not only solubilized the ligand and on the other side helped to coordinate the metal center, making it less sensitive to the change of acid concentration. Together with the ease of large-scale production, greener synthetic and purification procedures, high crystallinity, chemical stability, and superior extraction kinetics/performances, we believe that the current ligand design could inspire a renaissance in searching for new functional groups and coordination modes for efficient, high-acid-tolerant ligands and provide a clear step toward closing the nuclear fuel cycle.

## Results and Discussion

### Ligand Synthesis

**Figure 1:**
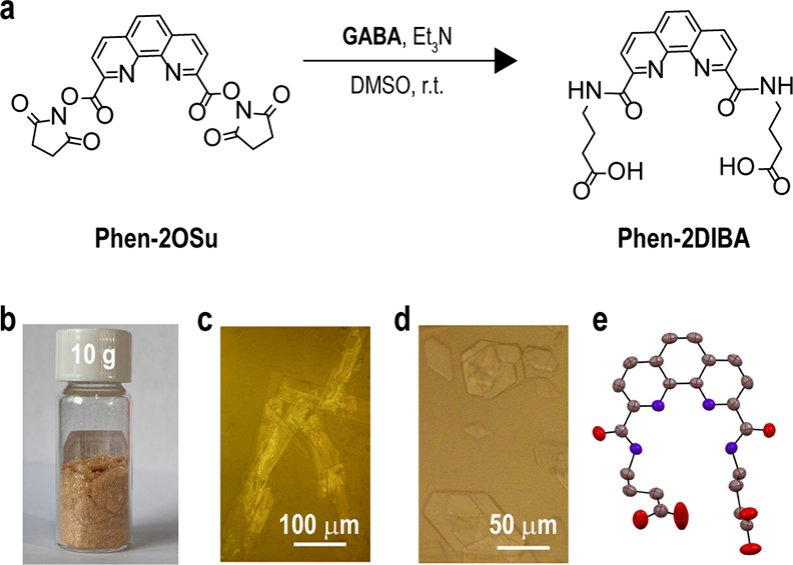
(a) Synthesis procedure for **Phen-2DIBA**. (b) Photograph of the crystal product after filtration with a 10 g scale. Bright field images of the as-synthesized **Phen-2DIBA** crystals (c) and crystals from a DMF/H_2_O mixture (d). (e) Single-crystal X-ray diffraction structure of the ligand in ellipsoid mode. Gray, blue, and red represent C, N and O, respectively.

The dibutyric acid functionalized phenanthroline diimide ligand (Figure 1a, hereafter referred as **Phen-2DIBA**) was synthesized following our previous report.^44^ Briefly, the *N*-hydroxysuccinimide activated phenanthroline dicarboxylic precursor was mixed with 4-aminobutyric acid in dimethyl sulfoxide (DMSO) at room temperature. A catalytic amount of triethylamine was added, and then the mixture was stirred overnight before pouring into water to quench the reaction. After acidification with hydrocholoride acid, belt-like crystals with a length of over a millimeter were readily afforded, indicating the high crystallinity of the ligand (Figure 1c). The whole synthetic procedure was simple, and no corrosive regent was used. An analytically pure product was afforded after simple filtration, which is straightforward for mass production. The chemical structure of the ligand was verified by ^1^H and ^13^C NMR and HRMS (Figures S1–S3). The *n*-butylamine modified ligand (named **Phen-2DIC4**, Figure S4) was also prepared for a direct comparison of the end group effect. Single crystals of **Phen-2DIBA** with suitable size and dimensions were grown from a DMF/H_2_O mixture. As shown in Figure 1e, the two flanked imides were oriented with N–H bonds pointing toward the phenanthroline cavity because hydrogen bonds formed between the two imide N–H and another imide oxygen from adjacent molecules (Figure S5 and Table S1). Although both ligands were barely soluble in water at room temperature, **Phen-2DIBA** could totally dissolve in HNO_3_ with a concentration higher than 1.25 M (5 mM ligand concentration, Figure S6, probably because of the protonation of the phenanthroline backbone). The solubility of **Phen-2DIBA** in HNO_3_ could be synergistically contributed by both carboxyl end groups and the protonation of the phenanthroline skeleton, as **Phen-2DIC4** remained insoluble in 1.5 M HNO_3_ under similar conditions (Figure S6). Also, **Phen-2DIBA** was stable in HNO_3_ (1.5 M) during a period of 1 week, as revealed by NMR data discussed in the NMR titration part.

### Solvent Extraction Behaviors

**Figure 2:**
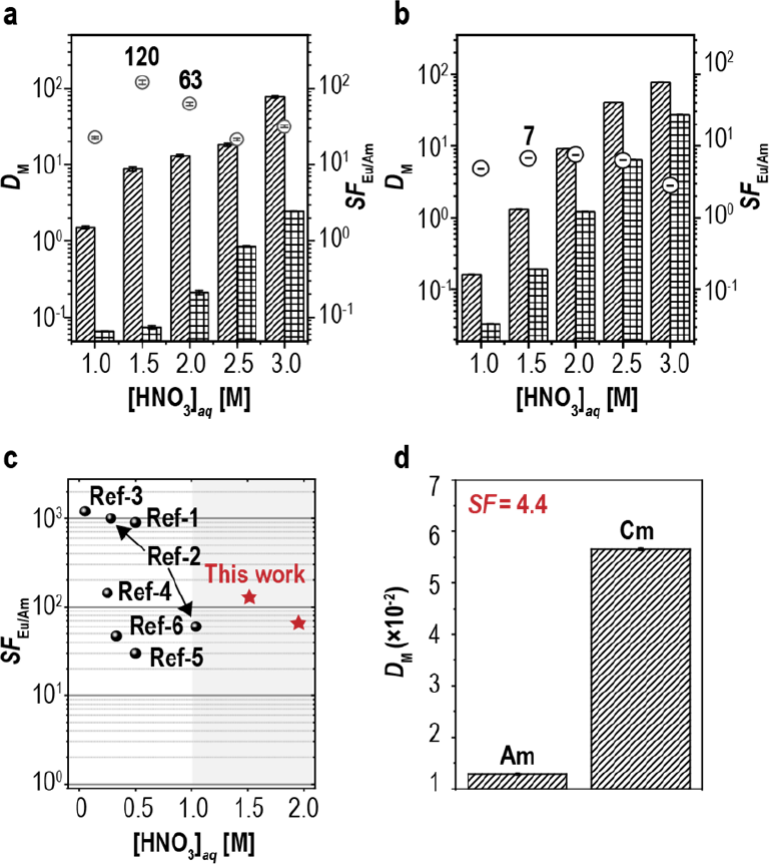
Distribution ratios (*D*_M_, bars with slashes for Eu(III) and gridded bars for Am(III)) and separation factors (SF, black circles) obtained in the extraction of Am(III) and Eu(III) by **TODGA** with (a) and without (b) **Phen-2DIBA** as a function of acid concentrations. Organic phase: **TODGA** (0.05 M) in dodecane. Aqueous phase: 5 mM **Phen-2DIBA** in HNO_3_ with NaNO_3_. The total concentration of NO_3_^–^ was fixed to 3 M. O/A = 1. Vortex shaker (50 Hz) for 30 min at 25 ± 1 °C. (c) Extraction performance comparison of the hydrophilic masking ligands reported in the literature with **Phen-2DIBA**. (d) Distribution ratios (*D*_M_) and separation factors (SF) obtained in the extraction of ^241^Am(III) and ^244^Cm(III) by **TODGA** in the presence of **Phen-2DIBA**. Organic phase: **TODGA** (0.05 M) in dodecane. Aqueous phase: 5 mM **Phen-2DIBA** in 1.5 M HNO_3_ with 1.5 M NaNO_3_. O/A = 1. Vortex shaker (50 Hz) for 30 min at 25 ± 1 °C.

The solvent extraction behaviors of **Phen-2DIBA** were demonstrated by using **Phen-2DIBA** as the aqueous masking agent in combination with **TODGA** in dodecane as the organic phase. The initial screening of the acid concentration gave satisfying results: **Phen-2DIBA** showed clear selectivity of Am(III) over Eu(III) with the best separation factor of about 50 in 1.5 M HNO_3_ (Figure S7). The control experiment of pure **TODGA** revealed that the observed Am(III) shielding effect was mainly contributed by the new hydrophilic ligand in the aqueous phase (Figure 2b). This unoptimized result outperformed the SF_Eu/Am_ value of 47 for **BTrzPhen** in 0.33 M HNO_3_^39^ and the SF_Eu/Am_ value of 30 for **EtOH-BPTD** in 0.5 M HNO_3_^38^ and was comparable to the SF_Eu/Am_ value of 60 for **TS-BTPhen2** in 1.04 M HNO_3_ (Figure 2c and Table S2).^34^ Indeed, this SF_Eu/Am_ value for **Phen-2DIBA** in highly concentrated HNO_3_ was comparably high even among the well-studied lipophilic phenanthroline diamide ligands (e.g., SF_Eu/Am_ value of 40 for **QL-DAPhen** in 2 M HNO_3_ and SF_Eu/Am_ value of 67 for **Et-Tol-DAPhen** in 1 M HNO_3_, Scheme S2).^17,18^

Nitrate ion strengths in the aqueous phase are known to affect the performance of both lipophilic and hydrophilic ligands; thus, we further investigated the effect of nitrate ion on the extraction process.^26,27^ As shown in Figure 2a, when NaNO_3_ was added, the distribution ratios of both Eu(III) and Am(III) increased by about one order of magnitude because of the reverse shifting of coordination equilibrium with a larger extended effect observed for Eu(III). The effect of nitrate ion strengths on the extraction performances of **Phen-2DIBA** was further demonstrated, and the results are given in Figure S8. The nitrate ion strength control further increased the SF_Eu/Am_ of **Phen-2DIBA** to 120, which was similar to that of 140 for **PyTri** (0.25 M HNO_3_)^28^ and 170 for **DS-Ph-DAPhen** (0.3 M HNO_3_)^27^ while at a much higher acidity (Table S2). Thanks to synergistic effects from both the preorganized phenanthroline skeleton and fast kinetics of **TODGA**, the coordination of **Phen-2DIBA** toward both Eu(III) and Am(III) reached equilibrium in about 5 min (Figures S9 and S10).^17,24^ Additionally, the Eu(III)/Am(III) separation performances of **Phen-2DIBA** were further demonstrated in the stripping (back-extraction) experiment as described in the *i*-SANEX process for direct comparison with the procedures reported in the literature^28^ (Note 1 in the Supporting Information, data given in Tables S3 and S4) and results similar to those described above were observed. Lastly, the high selectivity of **Phen-2DIBA** toward Am(III) was demonstrated by americium–curium separation, which was believed to be a more challenging process, as Am(III) and Cm(III) are adjacent actinides with nearly identical radii.^45^ Curium isotopes were short-lived, intensively radioactive, and strong neutron emitters which were proposed to be separated as early in the separation process as possible in order to develop a compact separation process.^37,39^ The equilibrium separation factor of **Phen-2DIBA** toward Cm(III) and Am(III) was determined to be around 4.4 (Figure 2d). To the best of our knowledge, this was among the best curium–americium separations in hydrophilic ligands.^27,28,30,34,36−39^ While the distribution ratios were quite low at about 0.055 for *D*_Cm(III)_ and 0.015 for *D*_Am(III)_, adding NaNO_3_ increased both *D*_Cm(III)_ and *D*_Am(III)_ by 1 order of magnitude to a similar extent (Figure S11). Considering the high selectivity of **Phen-2DIBA** in both Eu(III)/Am(III) and Cm(III)/Am(III) separation under high acidity, the fast extraction kinetics, the CHON-compatible nature, and the absence of buffer used in the separation process, **Phen-2DIBA** represented a leap forward toward a closed nuclear fuel cycle.

### Complexation Behaviors

**Figure 3:**
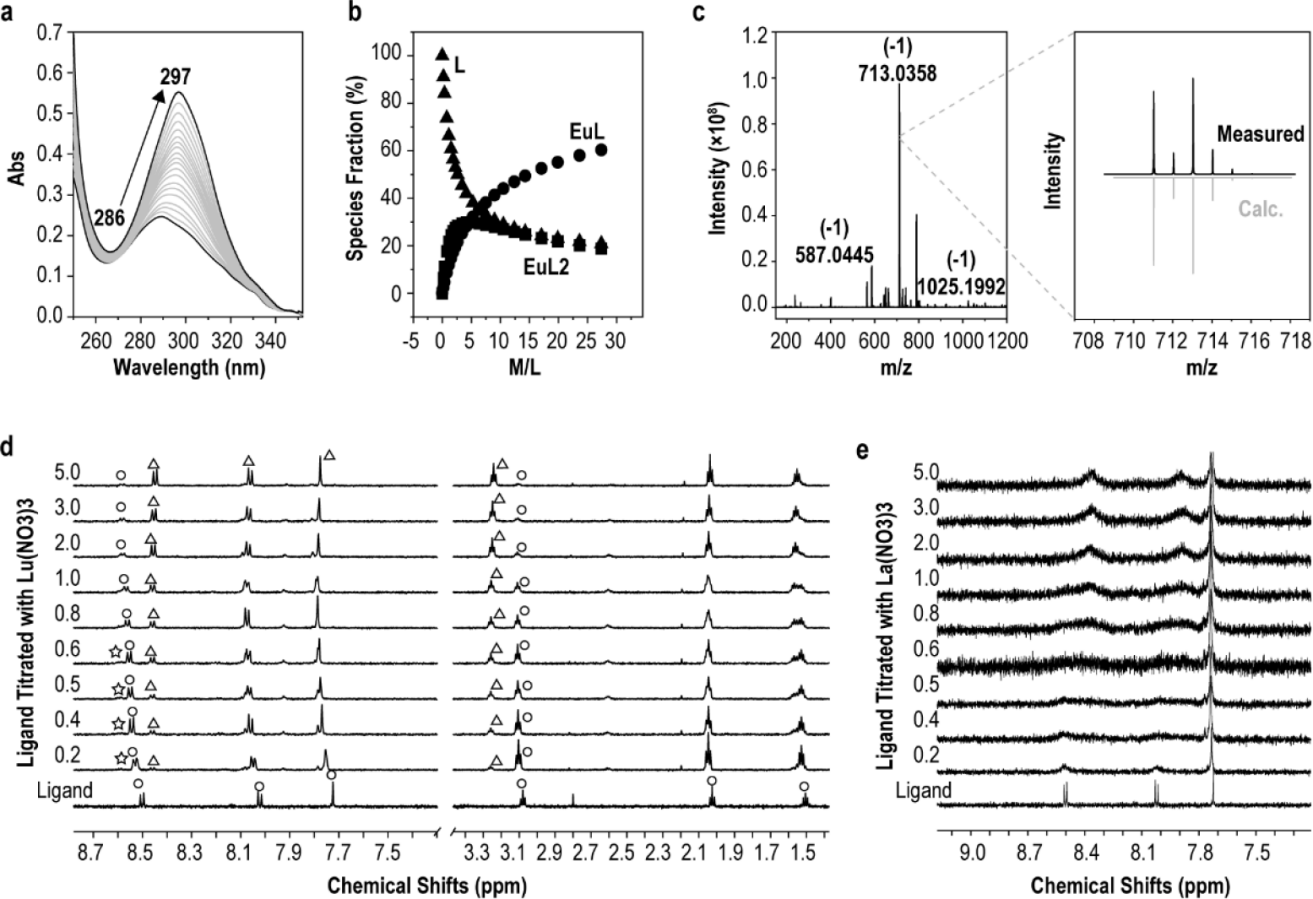
(a) UV–vis absorption spectra of titration of **Phen-2DIBA** with Eu(NO_3_)_3_ in 0.01 M HNO_3_ (0.1 M NaNO_3_). The ligand concentration was 0.01 mM. The 286 and 297 nm peaks showed the initial and final peak positions at the longer wavelength. (b) Species distribution of Eu(III) with **Phen-2DIBA** derived from (a). (c) Negative mode ESI-MS of **Phen-2DIBA** with an equivalent amount of Eu(NO_3_)_3_ in methanol. Enlarged spectra of the peak at *m*/*z* 713.0358 was given corresponding to Eu(III) = 1 species. Stacked ^1^H NMR spectra of **Phen-2DIBA** titrated with Lu(NO_3_)_3_ (d) and La(NO_3_)_3_ (e). Metal/ligand ratios are given on the left sides of each panel. Circles, triangles, and stars represent the ligand and 1:1 and 1:2 species, respectively. Experimental conditions: 5 mM **Phen-2DIBA** in 1.5 M DNO_3_/D_2_O titrated with 50 mM of the corresponding metal nitrate salts. The circles and triangles in (d) show the peaks originating from **Phen-2DIBA** and **Phen-2DIBA**/Lu(III) 1:1 species.

In order to obtain further information about the complexation behaviors of **Phen-2DIBA** with the trivalent metal ions in solution, UV–vis absorption spectroscopy titrations were conducted in 0.01 M HNO_3_ with ion strength controlled by 0.1 M NaNO_3_. With the addition of Eu(III) ions, the peak at round 286 nm for **Phen-2DIBA** gradually red-shifted to 297 nm with increasing absorption intensities, which is a common phenomenon indicating the existence of a metal–ligand interaction (Figure 3a).^7^ Data fitting with the *HypSpec* program gave the species evolution during the titration process.^46^ As shown in Figure 3b, both 1:1 and 1:2 metal/ligand species existed in solution with the dominant species being 1:1 (titration data in noncoordination solvent HClO_4_ and NaNO_3_, apparent stability constants for both titrations in HNO_3_ and HClO_4_ are given in Figure S12 and Table S5). These results were further confirmed by ESI-MS. The negative mode ESI-MS results in Figure 3c gave three main ligand-associated species located at *m*/*z* 587.0445 (−1), 713.0358 (−1) and 1025.1992 (−1), corresponding to [Eu(**Phen-2DIBA**-4H)]^−^ (587.0444), [Eu(**Phen-2DIBA**-2H)(NO_3_)_2_^–^]^−^ (713.0356), and [Eu(**Phen-2DIBA**-2H)_2_]^−^ (1025.1983) (full MS data are given in Figure S13 with species analysis in Table S6). Finally, the complexation of **Phen-2DIBA** with the trivalent metal ions was further explored by ^1^H NMR titrations under the same conditions used in the extraction experiment. La(III) and Lu(III) ions were used because they represented the largest radius differences in all Lns (closer to the case in Eu(III) and Am(III) separation) and also because of their diamagnetic nature. Titration data of **Phen-2DIBA** with both Lu(NO_3_)_3_ and La(NO_3_)_3_ are depicted in Figure 3d,e. With 0.2 equiv of Lu(III) added to 5 mM **Phen-2DIBA** in 1.5 M DNO_3_/D_2_O, three species of ligand and ligand/metal with ratios of 1:1 and 1:2 were clearly detected (Figure 3 and Figures S14–S16). Further increasing the metal ions resulted in the downfield shifting of the aromatic peaks (most obvious for the peak at around 8.5 ppm), which has been frequently reported in the literature and could be ascribed to the metal coordination reducing the electron densities of the ligand.^7,17,25,44,47,48^ After 5 equiv of Lu(III) was added, well-defined peaks corresponding to 1:1 species of **Phen-2DIBA**/Lu(III) dominated in the solution. A similar phenomenon was observed for the alkyl chain peak at around 3.1 ppm (Figure S16, corresponding to proton no. 5 on **Phen-2DIBA** as indicated by 2D H–H COSY in Figure S17). The NMR titration data were echoed by UV–vis titrations and ESI-MS results, all of which strongly supported the dominant species of 1:1 ligand/metal in the aqueous phase. Furthermore, when **Phen-2DIBA** was titrated with La(NO_3_)_3_ under the same conditions, only ostensibly upfield-shifted broad peaks were detected (Figure 3e and Figures S18 and S19). Typically, broad peaks in NMR spectra indicated multiple species coexisted in the solution and no well-defined species formed.^48^ La(III) and Lu(III) ions only differed in their atomic radius in the current case; the NMR titration results thus could be indirect evidence for the superior ion selectivity of **Phen-2DIBA**. Finally, it is worth pointing out that the stabilities of the ligand and La(III)- and Lu(III)-related species were adequately stable under high-acidity conditions (1.5 M HNO_3_) as indicated by NMR spectroscopy, and no decomposition or precipitation was detected during a time period of 1 week (Figures S20–S22).

### Coordination Mode Analysis

**Figure 4:**
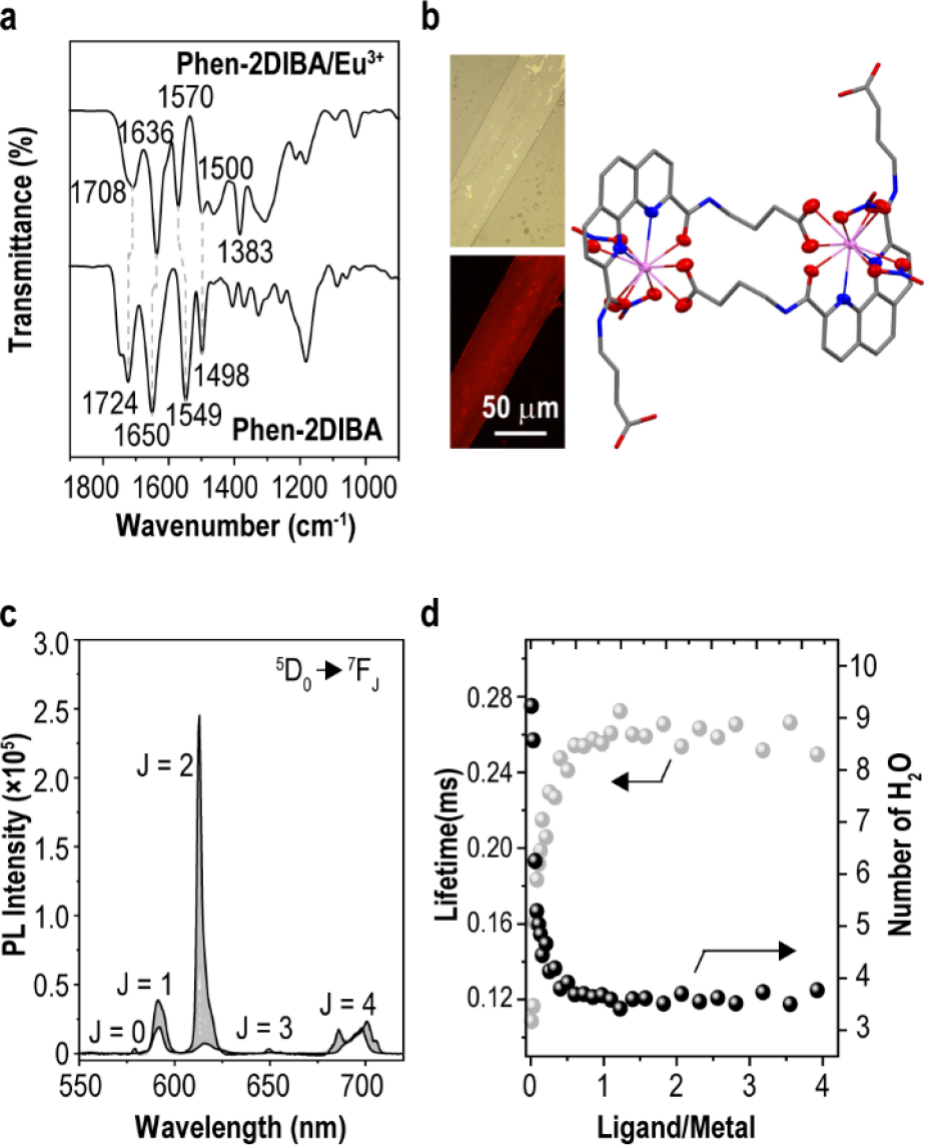
(a) IR spectra of **Phen-2DIBA** and **Phen-2DIBA**/Eu(III) (1:1) species with main peaks marked on the spectra. (b) Bright-field image of single crystal from the **Phen-2DIBA**/ Eu(III) (1:1) mixture (left top) and a photoluminescence image excited with 350 nm light (left bottom). The single-crystal X-ray diffraction structure of the **Phen-2DIBA**/Eu(III) (1:1) complex is given in capped sticks mode with the inner sphere of the Eu(III) coordination atoms labeled in ellipsoid mode. Gray, blue, red, and purple represented C, N, O, and Eu, respectively. TRLFS titrations of **Phen-2DIBA** to Eu(III) (c), lifetime monitored at 613 nm, and deduced water molecule number around the Eu(III) center during titration (d). Titration conditions: *C*_Ligand_/*C*_Eu(III)_ = 4 mM/1 mM, titrant **Phen-2DIBA** in 1.5 M HClO_4_, *I* = 1 M NaClO_4_, and 1.585 mL of titrant added. *V*_0_ = 1.6 mL.

The coordination modes of **Phen-2DIBA** with Eu(III) were elucidated by IR spectra and single-crystal X-ray diffraction. The characteristic IR peaks of **Phen-2DIBA** were definitively assigned according to literature reports^7,17,27,44^ and by comparison with the IR spectra of **Phen-2DIC4** (Figure S23). As given in Figure 4a and Figure S20, after coordinating with Eu(III) ions, the C=O (imide) peak at 1650 cm^–1^ shifted to 1636 cm^–1^ while the C=N peak (phenanthroline) at 1549 cm^–1^ shifted to 1570 cm^–1^, which was well consistent with that observed for hydrophilic **DS-Ph-DAPhen** (Full IR spectra are given in Figure S24; note that the peak at around 1380 cm^–1^ was from nitrate ion^49^).^27^ Moreover, an obvious shift was also detected for C=O stretching (from 1724 to 1708 cm^–1^) on the carboxylic group, which strongly indicated that the carboxylic groups were involved in the coordination process in a certain way. This has been commonly reported for Lns(III) complexes bearing carboxylic groups but is rarely seen in ligand design for Lns(III)/Ans(III) separation.^41−43^ To confirm this hypothesis, single crystals of **Phen-2DIBA**/Eu(III) complexes (ligand/metal ratio of 1:1) were grown from a concentrated solution of **Phen-2DIBA**/Eu(III) in a methanol/isopropanol mixture (v/v = 1/1, Note 2 in the Supporting Information). As depicted in Figure 4b, long beltlike crystals of **Phen-2DIBA**/Eu(III) were afforded which showed characteristic red emission of Eu(III) complexes under 350 nm excitation. Unexpectedly, the complexes adopted a very uncommon coordination mode of 2:2 ligand/metal architecture. Each metal center was coordinated by ten atoms: four from the **Phen-2DIBA** ligand (N and O), four from two nitrate cations (O), and the other two from the deprotonated carboxylic groups of another adjacent **Phen-2DIBA** ligand (O). Interestingly, both the **Phen-2DIBA** ligand and **Phen-2DIBA**/Eu(III) complex belonged to the monoclinic space group holding four **Phen-2DIBA** molecules in one unit cell (Figures S25–S27 and bond lengths in Table S7). The ensemble level purities of both **Phen-2DIBA** ligand and **Phen-2DIBA**/Eu(III) complexes were examined by powder X-ray diffraction (PXRD), and the results are given in Figure S28 and compared to the simulated results derived from single-crystal X-ray diffraction data. The overall matches of the two data sets indicated the structural similarities of the ensemble sample and the single-crystak X-ray diffraction results. The 10-coordinated architectures are common for Lns(III)/Ans(III) complexes, while, to the best of our knowledge, this is the first report on this kind of dimer-like two-metal–two-ligand coordination mode for Eu(III) complexes observed for hydrophilic Lns(III)/Ans(III) separation by a phenanthroline diimide ligand (detailed crystal data for both **Phen-2DIBA** ligand and Eu(III) complexes are summarized in Tables S8 and S9). To better understand the binding differences of the **Phen-2DIBA** ligand with Eu(III) and Am(III), we calculated the bond length on the optimized complex geometries with a simplified 1:1 architecture of [M(NO_3_)_3_L]. The results are discussed in Note 3 in the Supporting Information (Figure S29 and Table S10). The relatively longer Eu–N and shorter Eu–O bonds in comparison with Am–N and Am–O agreed well with the reported data for other hydrophilic ligands,^27^ indicating the softer nature of Ans(III).

### Discussion

With all of the above data in hand, we could now try to unravel the superior selectivity of **Phen-2DIBA** among both Lns(III)/Ans(III) and Ans(III)/Ans(III) in 1.5 M HNO_3_. The single-crystal structure in Figure 4b revealed an eight-oxygen, two-nitrogen coordination architecture around the metal center for **Phen-2DIBA** instead of the coordination of two tetradentate (*ONNO* or *NNNN*) ligands together with another nitrate cation (10-coordination)^24,50−52^ or other monodentate solvent molecules (9-coordination)^39,52,53^ as frequently reported for other phenanthroline diamide (or triazine) derivatives. This was easy to understand considering the preferable coordination of “hard” oxygen in comparison with “soft” nitrogen. In the case of most *ONNO*-tetradentate ligands, the commonly observed coordination modes could be an entropy-drove process: it is energetically easier to arrange three molecules (two tetradentate ligands with a nitrate cation) rather than four around the metal center (one tetradentate ligand with three nitrate cations). While this entropy-favored architecture was not stable, the inferior binding affinity of nitrogen in comparison with oxygen was reflected as the loss of the selectivity and extraction ability of the ligand under high acidity (protonation of ligand and the competition of *O*-coordination from nitrate cations). In the current situation, the carboxylic group helped to stabilize the coordination of the metal center while it did not contribute too much to the total entropy change by taking a dedicated bimolecular coordination architecture. Furthermore, the relatively hydrophobic environment in the bridging zone could partially prevent the approach of a competing cation, further increasing the complex stability. The final question remaining was whether the single-crystal structure in Figure 4b could represent the true coordination in the extraction process with high acidity, as only methanol/isopropanol was used to cultivate the crystals. To answer this question, time-resolved laser fluorescence spectroscopy (TRLFS) titrations were conducted, and the results are given in Figure 4c. With the addition of **Phen-2DIBA** into a solution of Eu(ClO_4_)_3_ in 1.5 M HClO_4_, the characteristic Eu(III) emission gradually increased. Excitation spectra were monitored at multiple peaks for Eu(III) (591 nm for ^5^D_0_ to ^7^F_1_ transition and 613 nm for ^5^D_0_ to ^7^F_2_) to confirm the origins of these Eu(III) emission peaks were from the sensitization of the **Phen-2DIBA** ligand (Figure S30). The lifetimes of the 613 nm peaks were also monitored and plotted as a function of ligand/metal ratios (Figure 4d). Increasing the ligand/metal ratios prolonged the average lifetimes of the system, and a plateau was reached at around 1, which was consistent with our previous observation of predominant 1:1 species in the extraction process. The calculated number of water molecules around the Eu(III) center during titration decreased from nine in the initial Eu(ClO_4_)_3_ solution to four at the end of the titration (Note 4 in the Supporting Information, Figure S31, and Table S11). This matched well with the single-crystal data as in Eu(ClO_4_)_3_ solution, Eu(III) could be surrounded by nine water molecules, and after an excess amount of **Phen-2DIBA** was added, the proposed complexes similar to dimer-like structure in Figure 4b dominated, giving only four water molecules left around the metal centers (replacing the two nitrates in Figure 4b).

## Conclusions

Partitioning Lns(III) and Ans(III) is one of the most challenging hydrometallurgical separations known by far due to their chemical/physical similarities. Delicately designed hydrophilic ligands were believed to be an efficient way to overcome the massive amount of hazardous organic solvent used in the liquid–liquid extraction process and stood as a new frontier in this old area. However, currently reported hydrophilic ligands suffer from harsh preparation conditions, inferior extraction performances, limited available chemical structures, and incapable extraction under high acidity. In this article, we reported a simple yet efficient carboxylic group modified hydrophilic phenanthroline-based tetradentate *N*,*O*-ligand which displayed superior Lns(III)/Ans(III) and Ans(III)/Ans(III) separation capabilities under high acidity (over 1.5 M HNO_3_). An SF_Eu/Am_ value of 120 and SF_Cm/Am_ value of 4.4 were observed in 1.5 M HNO_3_ when the new hydrophilic ligand was used as a masking agent in combination with **TODGA** in dodecane as the organic extracting phase. A metal/ligand ratio of 1 was dominant in the solution extraction process as revealed by UV–vis absorption, NMR titrations, and ESI-MS. IR and single-crystal structures further confirmed the bifunctional role of carboxylic groups. Overall, we demonstrated through dedicated ligand design and crystal engineering that highly efficient and acid-tolerant Lns(III)/Ans(III) and Ans(III)/Ans(III) could be fulfilled.

## Supporting Information Available

Materials and methods, experimental procedures, supplementary solvent extraction details and back-extraction discussions, full ESI-MS and detailed species assignments, full NMR spectra titrations and stability tests, detailed crystal data and DFT calculation methods. and discussions of results (PDF)
Transparent Peer Review report available (PDF)
