# Supplementary material for: A Simple yet Efficient Hydrophilic Phenanthroline-Based Ligand for Selective Am(III) Separation under High Acidity
Source: ACS Cent Sci. 2023 Jul 14;9(8):1642–9. doi: 10.1021/acscentsci.3c00504 (PMC10451031; doi:10.1021/acscentsci.3c00504)
Supplement: Supplementary file 1 — oc3c00504_si_001.pdf [file oc3c00504_si_001.pdf]

Supporting Information for:

A Simple yet Efficient Hydrophilic Phenanthroline-Based Ligand for Selective Am(III) Separation under High Acidity

Deshun Tian,[a],[b],§ Yaoyang Liu,[c],§ Yu Kang,[a],§ Yue Zhao,[a],[d] Pengcheng Li,*[b] Chao Xu,*[c] and Li Wang*[a]

---

[a] D. Tian, Y. Kang, Y. Zhao, Prof. L. Wang
Department of Chemistry, Capital Normal University
Haidian District, Beijing, 100048 China
E-mail: pengchengli@wit.edu.cn; xuchao@tsinghua.edu.cn; liwang862011@gmail.com

[b] D. Tian, Prof. P. Li
Institute of Materials for Optoelectronics and New Energy, Hubei Key Laboratory of Plasma Chemistry and Advanced Materials, School of Materials Science and Engineering, Wuhan Institute of Technology
Wuhan, Hubei, 430205 China

[c] Dr. Y. Liu, Prof. C. Xu
Institute of Nuclear and New Energy Technology, Tsinghua University
Haidian District, Beijing, 100084, China

[d] Y. Zhao
CAS Key Laboratory of Green Process and Engineering, State Key Laboratory of Biochemical Engineering, Institute of Process Engineering, Chinese Academy of Sciences
Haidian District, Beijing, 100190, China

§ Deshun Tian, Yaoyang Liu and Yu Kang contribute equally to this work

Materials and characterizations. All chemicals and ultradry solvents were purchased from Energy Chemical Inc. and used as received unless otherwise stated. **TODGA** was obtained from Qingdao Beitwall Technology Co., Ltd. and used without further purification. Milli-Q water was used for all the experiments. $Eu(NO_3)_3 \cdot 6H_2O$ was purchased from Aladdin with purity of 99.99%. $Eu(ClO_4)_3$ for spectrophotometric titration experiment was prepared by dissolving Eu_2O_3 into perchloric acid, and Eu_2O_3 is provided by Sigma-Aldrich. Stock solutions of radioactive tracers ^{244}Cm, ^{241}Am and $^{152,154}Eu$ was supplied by Institute of Nuclear and New Energy Technology (INET). *Caution: ^{244}Cm, ^{241}Am and $^{152,154}Eu$ are highly radioactive and radiotoxic isotopes and may impose serious health threats. And the relevant experiments were performed in a radiological facility dedicated to studies on transuranic elements.* Bis(2,5-dioxopyrrolidin-1-yl) 1,10-phenanthroline-2,9-dicarboxylate was prepared following the literature procedures.[1]

Nuclear Magnetic Resonance spectroscopy (NMR) was collected on a Brucker-600 spectrometer in deuterated dimethylsulfoxide (DMSO-d_6) with tetramethylsilane (TMS, 0.03% V/V) as the internal standard. High resolution mass spectrometric (HRMS) analyses were performed on a 12 T Solarix MALDI-FT-ICR MS (Bruker Daltonics). Fourier transform infrared (FT-IR) spectroscopy was performed on a Brucker Tensor 27 spectrometer; data were recorded in the range of 500 to 4000 cm^{-1} with a resolution of 4 cm^{-1}. X-ray diffraction studies were conducted on a Rigaku Synergy-R with Cu Kα radiation (1.5406 Å). The φ scan was applied for empirical absorption corrections. Cell parameters were calculated by global refinement of the positions of all collected reflections. The direct method was used to solve the crystal structures, and a full matrix least-squares technique based on F^2 was applied to refine this structure using the SHELXL 97 program. All hydrogen atoms were refined isotropically while all nonhydrogen atoms were done anisotropically. Powder X-ray diffraction (XRD) spectra were obtained using a Brucker-AXS D8 series 2 diffractometer, set to a Bragg Brentano Parafocussing Geometry. A Cu-Kα source generated X-rays at 40 kV at room temperature. The monochromated X-rays passed through a 2 mm exit slit and an automatic divergence slit of 0.2° onto the sample. Intensities were collected between 2θ of 5° and 50° on a Braun position sensitive detector (0.010° and 7.5 seconds per step). UV-vis absorption spectra were collected on a Shimadzu 2600 spectrometer with 1-cm cuvettes at 25 °C. Absorbance in the range of 250 to 450 nm were recorded with scan speed of 1200 nm/min and sampling interval of 0.5 nm. Photoluminescence spectra (PL) and photoluminescence excitation spectra (PLE) were recorded on a Hitachi F-4500 spectrometer with excitation of 300 nm. The excitation and emission slits were kept at 5 nm. PL titration and luminescence lifetimes were recorded on an Edinburgh FLS-1000 spectrophotometer equipped with a 450 W ozone-free xenon arc lamp. A pulsed microsecond xenon lamp with a power of 150 W and a pulse width of ~1 μs was used

as the excitation source. The decay data were analyzed using the software package installed on the Edinburgh FLS-1000 spectrophotometer, and the goodness of fit was assessed by minimizing the reduced function, χ^2, and visually inspecting the weighted residuals.

Solvent Extraction. The carboxylic-group containing ligand **Phen-2DIBA** showed very limited solubility in water. When acid was used, the solubility of **Phen-2DIBA** increased dramatically. As shown in Figure. S6, 1M HNO_3 could largely dissolve **Phen-2DIBA** (5 mM) while the solution appeared to be blurry (the solution turned clear when heated). Further increasing the acidity of the aqueous solution to 1.25 M HNO_3, totally clear solution was afforded (5 mM **Phen-2DIBA**). The general protocol for solvent extraction was as following: 0.05 M **TODGA** in dodecane was used as organic phase; aqueous phase was prepared by dissolving **Phen-2DIBA** in HNO_3 (1-3 M for acid concentration screening experiment and 1.5 M was used for other experiment to ensure that true solution was used). Tracer amount of ^{241}Am(III), ^{152,154}Eu(III) and ^{244}Cm was also added into the aqueous solution. 0.5 mL of both aqueous and organic phases were contacted in closed glass tubes. Once contacted, the mixture was vigorously shaken with vortex shaker for 30 minutes at 25 ± 1°C controlled with a water bath. After equilibrium, the two phases were separated by centrifugation at 3000 r/min for 2 minutes. Aliquots were subsampled and analyzed, the relative concentrations of ^{241}Am and ^{152,154}Eu(III) (or ^{244}Cm) in aqueous phases before and after extraction were measured using Liquid Scintillation Spectrometer (Quantulus 1220, PerkinElmer). The distribution ratio (*D*) was calculated by the ratio between the concentration (radioactivity counts per unit volume) in the organic phase and in the aqueous phase. The separation factors (*SF*) was determined by the ratio of distribution ratios of ^{152,154}Eu (III) (or ^{244}Cm) to ^{241}Am(III).

UV-vis absorption spectra titration. Absorption spectra titration was done on a Shimadzu 2600 spectrometer with 1-cm cuvettes at 25 °C. The stability constants of Eu(III) with **Phen-2DIBA** in both HNO_3 and $HClO_4$ at 298 K were calculated using the nonlinear regression program *HypSpec*.[2] For nitrate media (I = 0.1M Et_4NNO_3), 1.6 mL of 0.01 mM ligand in 0.01 M HNO_3 solution was placed in a 10 mm quartz cuvette. Then 0.3 mM Eu(III) in 0.01 M HNO_3 was gradually added into the cuvette. The absorption was monitored in the range of 250−350 nm. For perchlorate media (I = 0.1M $NaClO_4$), 0.3 mM Eu(III) in 0.01 M $HClO_4$ was added into a 0.01 mM ligand in 0.1 M $HClO_4$ solution, and the absorption was monitored in the range of 200 - 350 nm. The preliminary kinetic experiment results show that the complexation reactions could reach the equilibrium state within a few minutes, so the absorption spectra change was monitored after 5 minutes with each aliquot added.

PL and excitation spectra. PL and excitation spectra were collected on a Hitachi F-4500 spectrometer in 1-cm cuvette. 0.05 M **Phen-2DIBA** in 0.01 M HNO_3 with/without 30 eq.

$Eu(NO_3)_3 \cdot 6H_2O$ in 0.01 M HNO_3 were studied to show the origins of the well-defined multiple emission peaks.

Time-resolved laser fluorescence spectroscopy (TRLFS) titrations. PL and lifetime titration of Eu(III) were recorded on an Edinburgh FLS-1000 spectrophotometer equipped with a 450 W ozone-free xenon arc lamp. 1.6 mL of 1.0 mM $Eu(ClO_4)_3$ in 1.5 M $HClO_4$ (with 1 M $NaClO_4$) solution was placed in 1-cm cuvette. Then 4.0 mM of **Phen-2DIBA** in the same solvent was added in gradually ascent manner. The luminescence emission spectra were monitored in the wavelength range of 550−720 nm (0.5 nm per step, 3 nm bandwidth) by excitation at 394 nm (2 nm bandwidth). The lifetimes of the emission peaks located at 613 nm, corresponding to $^5D_0$ to $^7F_2$ transition of Eu(III) during the titration were also measured. A pulsed microsecond xenon lamp with a power of 150 W and a pulse width of ~1 μs was used as the light source. The decay data were analyzed using the software package installed on the Edinburgh FLS-1000 spectrophotometer, and the goodness of fit was assessed by minimizing the reduced function, χ^2, and visually inspecting the weighted residuals.

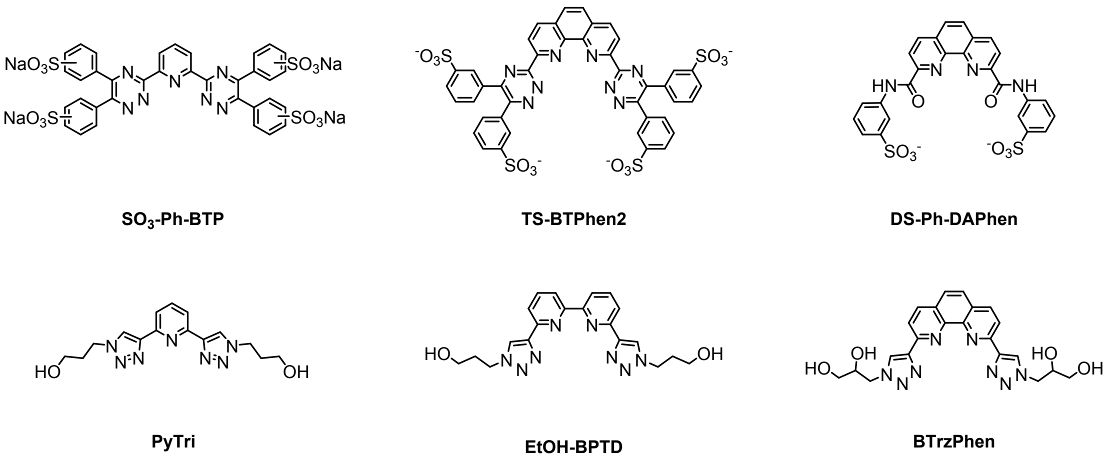

Scheme S1. Chemical structures for hydrophilic Eu(III)/Am(III) separation agents mentioned in the main text.

Ligand synthesis. Bis(2,5-dioxopyrrolidin-1-yl) 1,10-phenanthroline-2,9-dicarboxylate was synthesized according to our previous publication.[1] **Phen-2DIC4** was also prepared for direct comparison and data analysis.

Synthesis of 4,4'-((1,10-phenanthroline-2,9-dicarbonyl)bis(azanediyl))dibutyric acid (**Phen-2DIBA**)

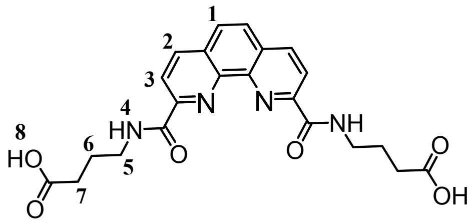

To a solution of bis(2,5-dioxopyrrolidin-1-yl) 1,10-phenanthroline-2,9-dicarboxylate (11.5 g, 25 mmol) in 100 mL DMSO was added 4-aminobutyric acid (6.4 g, 62.5 mmol, 2.5 eq.), then 0.5 mL of Et_3N was added to catalyze the reaction. The mixture was stirred at room temperature for 12 hours before water was added to precipitate the product. The final product was collected by filtration and dried in air. The product was collected as off-white crystals after recrystallization from the DMSO/H_2O mixture at 0 °C with a yield of 85% (9.3 g). ^{1}H NMR (600 MHz, DMSO-d_6) δ 12.08 (s, broad, 2H), 9.44 (t, J = 5.9 Hz, 2H), 8.73 (d, J = 8.2 Hz, 2H), 8.45 (d, J = 8.2 Hz, 2H), 8.18 (s, 2H), 3.50 (q, J = 6.7 Hz, 4H), 2.38 (t, J = 7.2 Hz, 4H), 1.92 (p, J = 7.1 Hz, 4H). ^{13}C NMR (151 MHz, DMSO-d_6) δ 174.95, 164.43, 150.22, 144.21, 138.68, 130.69, 128.36, 121.52, 39.18, 31.93, 25.08. HRMS m/z: $[C_{22}H_{23}N_4O_6]^+$ ($[M+H]^+$), calculated for 439.1613, found 439.1615.

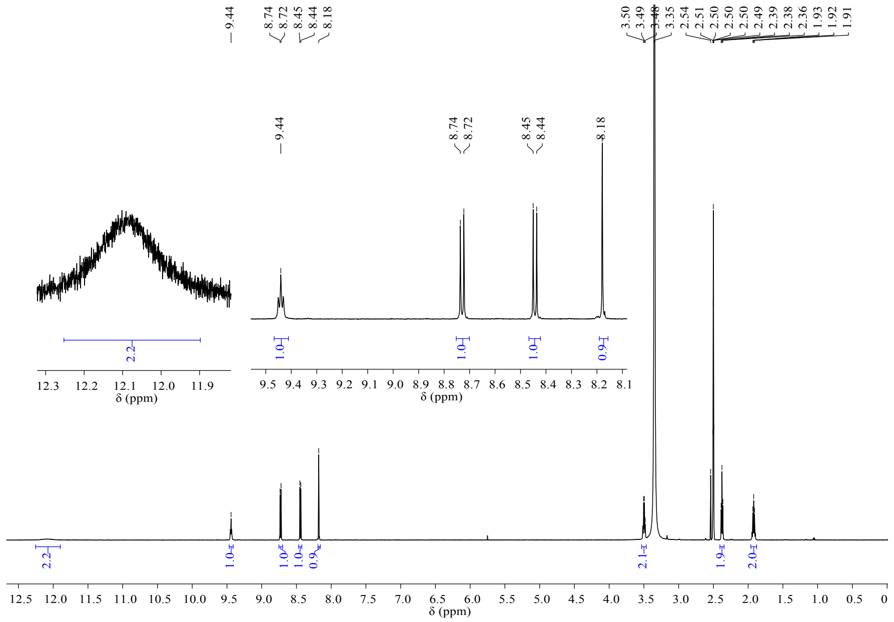

Figure S1. ^{1}H NMR spectrum of **Phen-2DIBA** in DMSO-D_6.

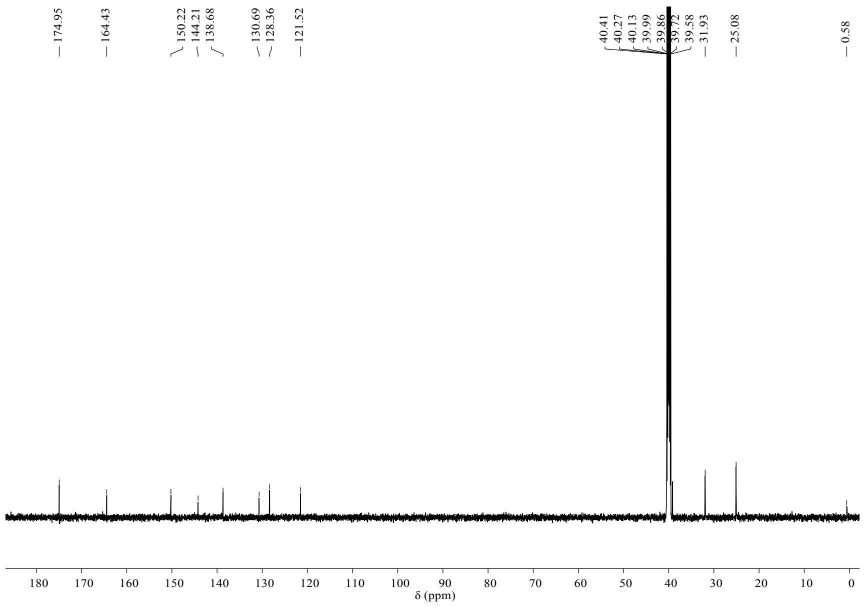

Figure S2. ^{13}C NMR spectrum of **Phen-2DIBA** in DMSO-D_6.

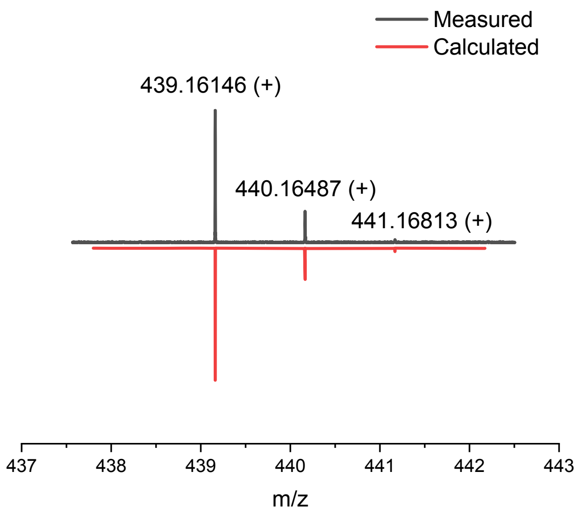

Figure S3. Measured and calculated MALDI-TOF-MS for **Phen-2DIBA**.

Synthesis of N[2],N[9]-dibutyl-1,10-phenanthroline-2,9-dicarboxamide (**Phen-2DIC4**)

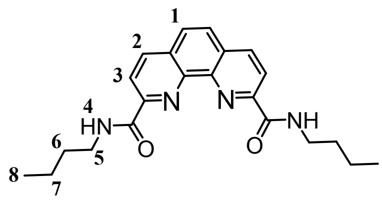

Similar procedure as the synthesis of **Phen-2DIBA** was followed. *n*-Butylamine was used and the final product was afforded as pale gray solid after filtration with a yield of 80%. ^{1}H NMR (600 MHz, DMSO-d_6) δ 9.42 (t, J = 5.9 Hz, 2H), 8.74 (d, J = 8.2 Hz, 2H), 8.45 (d, J = 8.2 Hz, 2H), 8.18 (s, 2H), 3.41 (m, 4H), 1.59 (m, 4H), 1.38 (m, 4H), 0.92 (t, J = 7.4 Hz, 6H).

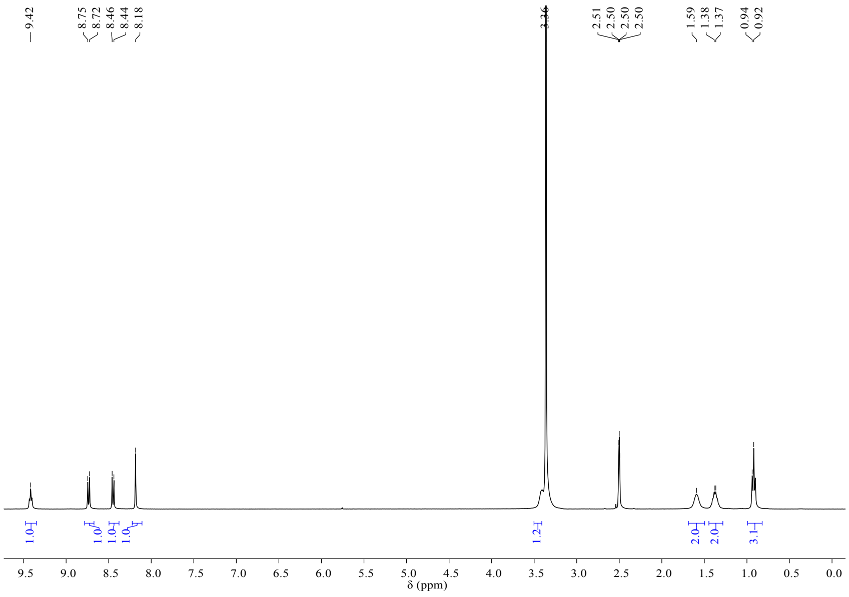

Figure S4. ^{1}H NMR spectrum of **Phen-2DIC4** in DMSO-D$_6$.

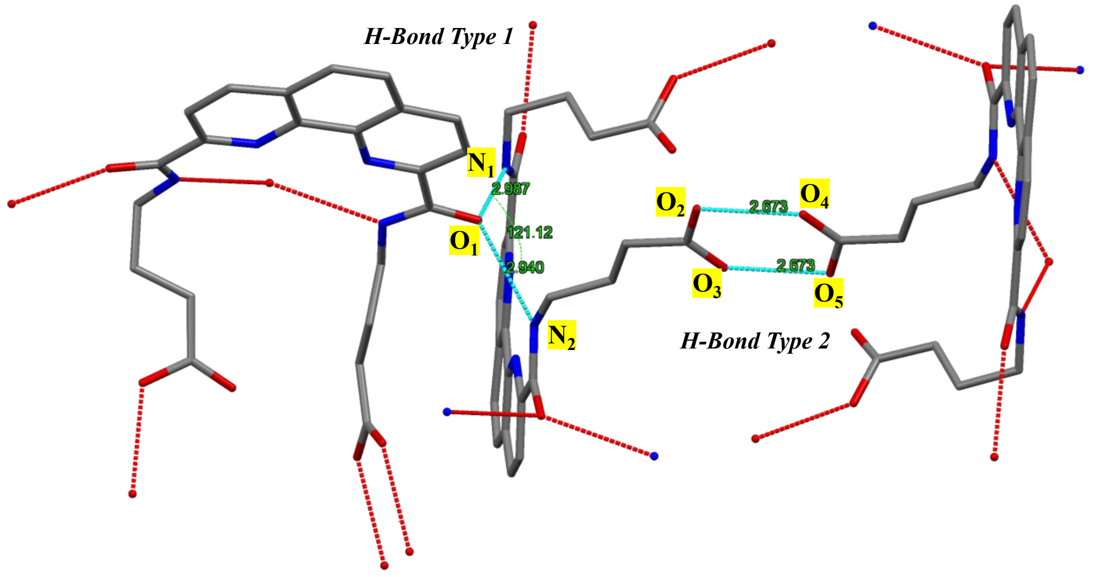

Figure S5. Molecular packing of **Phen-2DIBA** shows the hydrogen bonds formed between one oxygen atom and two N-H from another molecule. Two types of hydrogen bonds exist in the crystal, the second one forms between carboxylic groups. The distances shown on the figure give the bond lengths and angles between nonhydrogen atoms involved in the hydrogen bonds.

Table S1 Summary of hydrogen bonds in the crystal of **Phen-2DIBA**

Hydrogen Bond Type	Bonds	Distances	Angles
Type 1 (imide N-H to imide CO)	N_1-H-O_1	0.880; 2.259	140.01
	N_2-H-O_1	0.880; 2.261	133.91
Type 2 (carboxylic O-H to CO)	O_2-H-O_4	0.840; 1.837	172.52
	O_3-H-O_5	1.837; 0.840	172.52

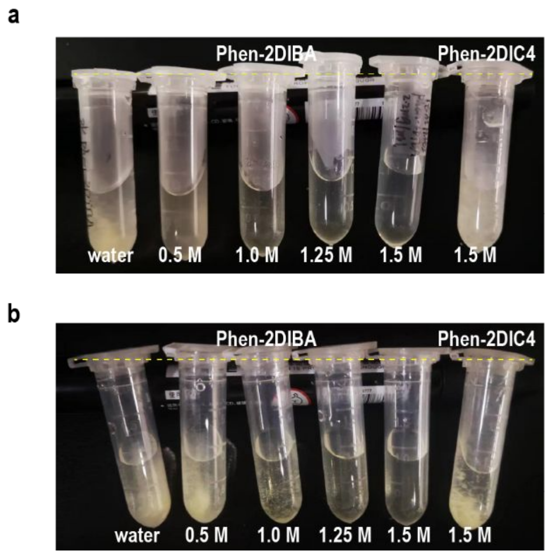

Figure S6. Photographs showing the solubility of both **Phen-2DIBA** and **Phen-2DIC4** in different concentration of HNO_3: (a) heated at 80 °C for 5 minutes; (b) after cooling down to room temperature. Ligand concentration was 5 mM for both **Phen-2DIBA** and **Phen-2DIC4**.

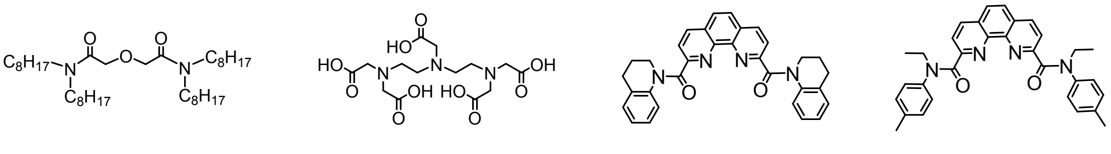

TODGA **DTPA** **QL-DAPhen** **Et-Tol-DAPhen**

Scheme S2. Other ligands mentioned in the main text. Molecular structures of *N,N,N′,N′*-tetraoctyl diglycolamide (**TODGA**), diethylenetriaminepentaacetic acid (**DTPA**), **QL-DAPhen** and **Et-Tol-Phen**.

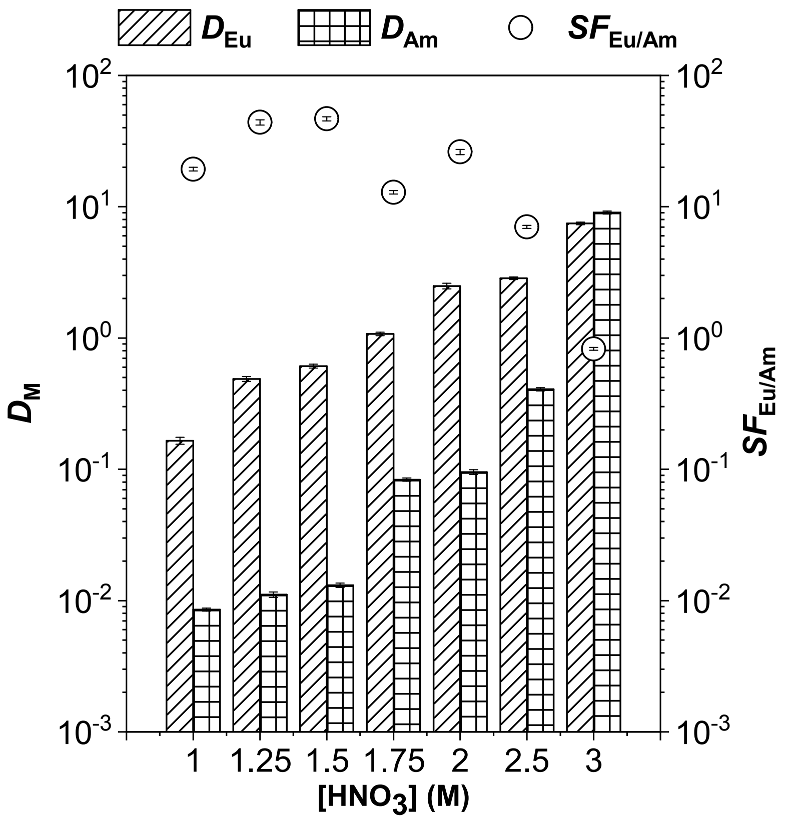

Figure S7. Extraction of Am(III) and Eu(III) by **TODGA** in the presence of **Phen-2DIBA** as a function of acid concentrations. Organic phase: **TODGA** (0.05 M) in dodecane. Aqueous phase: 5 mM **Phen-2DIBA** in different concentrations of HNO_3. O/A=1; Vortex shaker (50 Hz) for 30 minutes at 25±1°C.

Table S2. Extraction performance comparison of literature reported hydrophilic masking ligands

Ligands	$SF_{Eu/Am}$	$SF_{Cm/Am}$	$[HNO_3]$ (M)	Extraction conditions	References
**SO_3-Ph-BTP (Ref1)**	~900	NA	0.5	[a]OP: 0.2 M **TODGA** in 5% 1-octanol/TPH (a French kerosene);<br>[b]AP: 18 mM **SO_3-Ph-BTP** in 0.5 M HNO_3	3
**TS-BTPhen2 (Ref2)**	~200;<br>~60	~3.6	~0.7;<br>~1.04	OP: 0.2 M **TODGA** in 5% 1-octanol/kerosene;<br>AP: 10 mM **TS-BTPhen2** in ca. 0.7 M HNO_3	4, 5
**DS-Ph-DAPhen (Ref3)**	~1200;<br>~170	NA	~0.05;<br>~0.3	OP: 0.03 M **TODGA** in kerosene;<br>AP: 1 mM **DS-Ph-DAPhen** in ca. 0.05 M HNO_3 containing 1 M $NaNO_3$	6
**PyTri (Ref4)**	~140	NA	0.25	OP: 0.2 M **TODGA** in 5% 1-octanol/kerosene;<br>AP: 100 mM **PyTri** in ca. 0.25 M HNO_3	7
**EtOH-BPTD (Ref5)**	~30	~2	0.5	OP: 0.1 M **TODGA** in 5% 1-octanol/TPH (a French kerosene);<br>AP: 20 mM **EtOH-BPTD** in 0.5 M HNO_3	8
**BtrzPhen (Ref6)**	~47	~2.5	0.33	OP: 0.2 M **TODGA** in 5% 1-octanol/kerosene;<br>AP: 10 mM **BTrzPhen** in 0.33 M HNO_3	9
**Phen-2DTBA**	~120	~4.4	1.5	OP: 0.05 M **TODGA** in dodecane;<br>AP: 5 mM **Phen-2DTBA** in 1.5 M HNO_3 containing 1.5 M $NaNO_3$	This work

[a] Organic phase. [b] Aqueous phase.

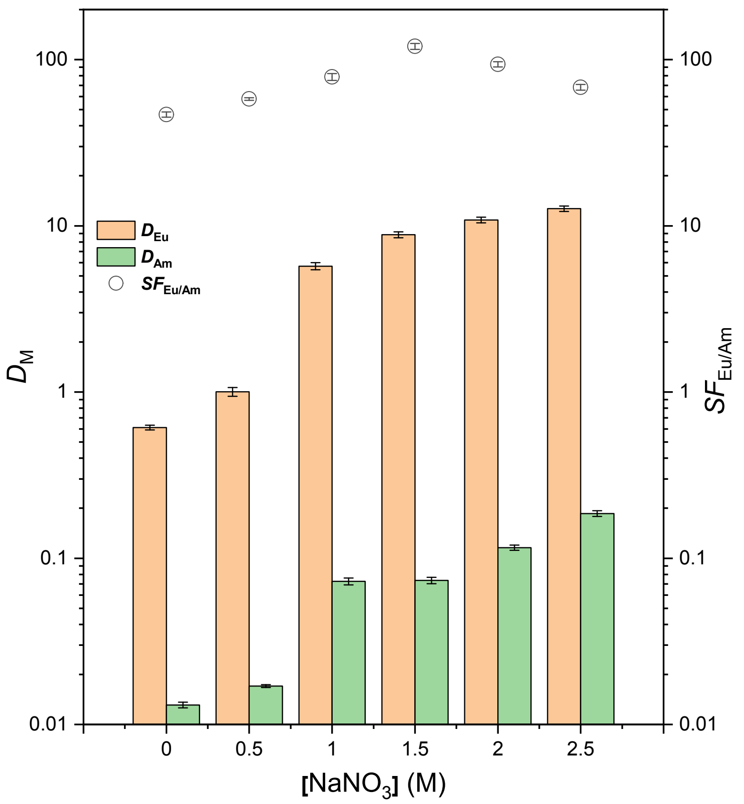

Figure S8. Extraction of Am(III) and Eu(III) by **TODGA** in the presence of **Phen-2DIBA** as a function of $NaNO_3$ concentrations. Organic phase: **TODGA** (0.05 M) in dodecane. Aqueous phase: 5 mM **Phen-2DIBA** in 1.5 M HNO_3. O/A=1; Vortex shaker (50 Hz) at 25±1°C.

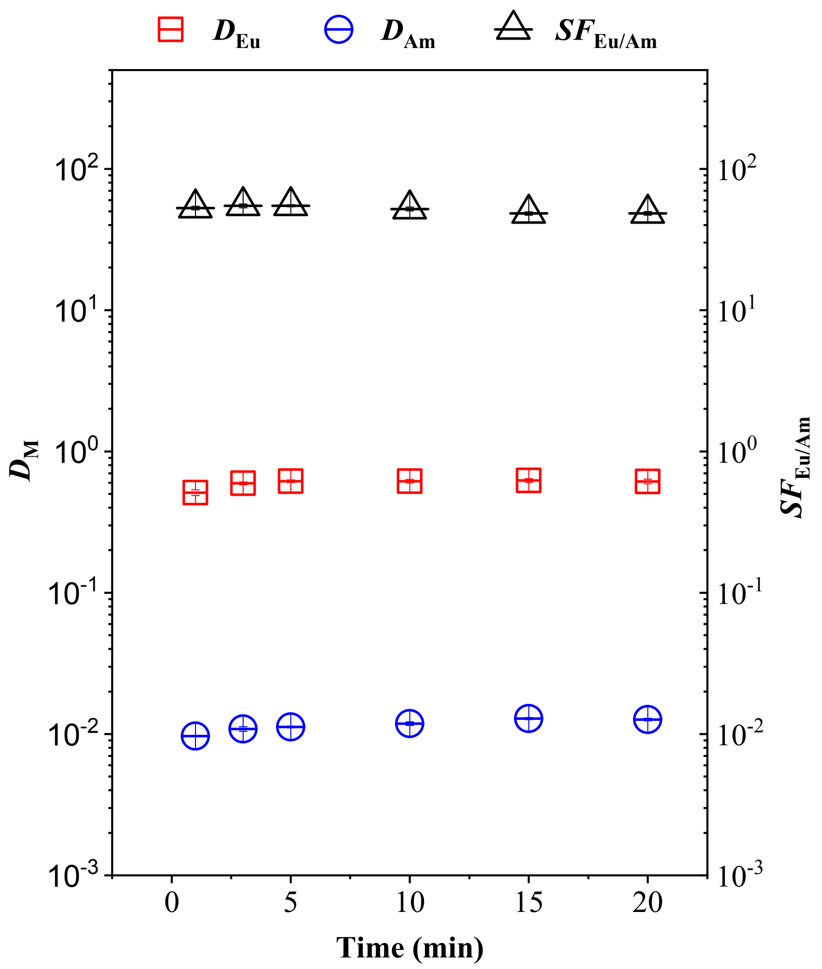

Figure S9. Extraction of Am(III) and Eu(III) by **TODGA** in the presence of **Phen-2DIBA** as a function of time. Organic phase: **TODGA** (0.05 M) in dodecane. Aqueous phase: 5 mM **Phen-2DIBA** in 1.5 M HNO_3. O/A=1; Vortex shaker (50 Hz) at 25±1°C.

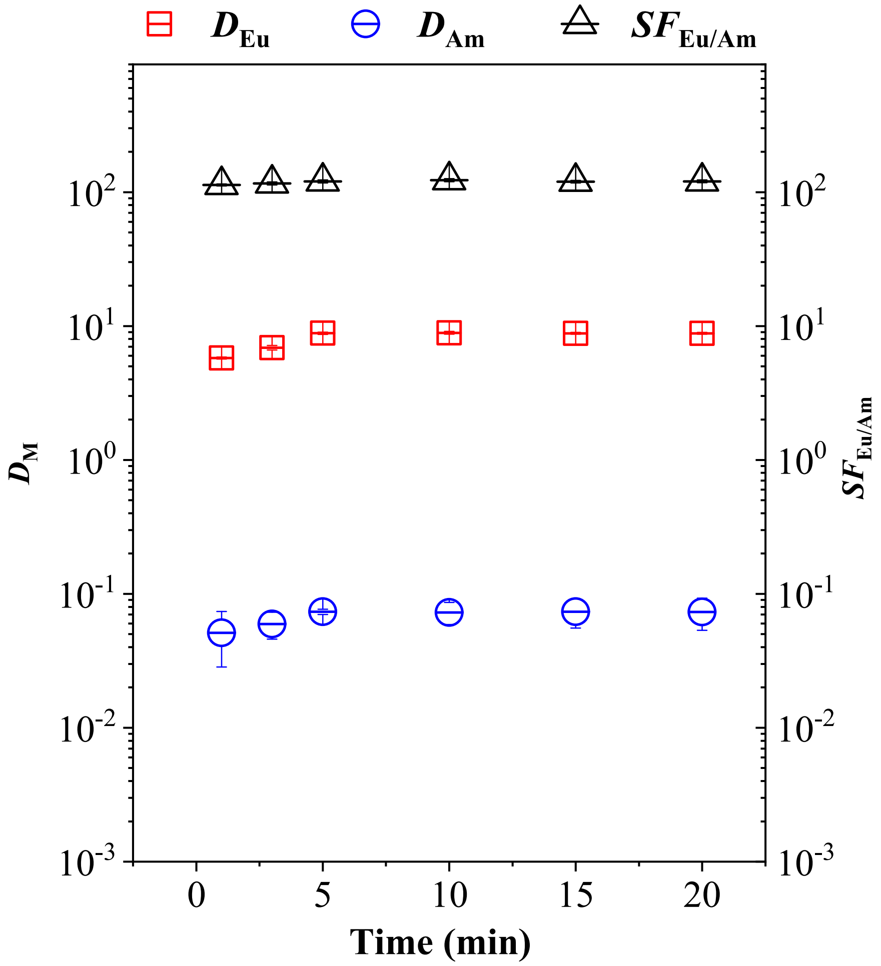

Figure S10. Extraction of Am(III) and Eu(III) by **TODGA** in the presence of **Phen-2DIBA** as a function of time. Organic phase: **TODGA** (0.05 M) in dodecane. Aqueous phase: 5 mM **Phen-2DIBA** in 1.5 M HNO_3 with 1.5 M $NaNO_3$. O/A=1; Vortex shaker (50 Hz) at 25±1°C.

Supporting Information Note 1.

There were now mainly two different approaches using hydrophilic ligands for the separation of Lns(III) and Ans(III): the TALSPEAK process developed in the USA and hot tested in Sweden and *i*-SANEX process developed by French CEA and investigated in the European ACSEPT project.[10] The former one used the combination of non-selective extraction agents such as **TODGA** together with aqueous masking agents (water-soluble selective ligands), in which the masking agents selectively "held" certain metal ions to assist the extraction of **TODGA**, given the overall separation. Most of the literature hydrophilic ligands were reported

with this process.[3-6, 8, 9, 11-14] In the second case, both Lns(III) and Ans(III) were coextracted by **TODGA**, then “stripping” agents were introduced to selectively back-extract certain metal ions, leaving the rest metal ions in the organic phase, given the overall separation.[7, 15] To further demonstrate the stripping performance of the current reported **Phen-2DIBA** ligand, the following stripping (back-extraction) experiments were conducted:

Experiment 1: Effect of **Phen-2DIBA** ligand concentrations on stripping of Am(III) from **TODGA**-based organic phases.

0.05 M **TODGA** in dodecane as organic phase (0.5 mL) were contacted with aqueous phase containing tracer amount of ^{241}Am(III) and ^{152,154}Eu(III) (0.5 mL, 3 M HNO_3) in closed glass tube. The mixture was vigorously shaken with vortex shaker for 30 minutes at 25 ± 1°C controlled with a water bath. After equilibrium, the two phases were separated by centrifugation at 3000 r/min for 2 minutes. The collected organic phases were contacted with aqueous phases containing different concentrations of stripping agents (1, 3, 5 mM of **Phen-2DIBA** in 1.5 M HNO_3 and 1.5 M $NaNO_3$). Same mixing and phase separation were repeated before aliquots were subsampled and analyzed, the relative concentrations of ^{241}Am and ^{152,154}Eu(III) in aqueous phases before and after extraction were measured using Liquid Scintillation Spectrometer (Quantulus 1220, PerkinElmer). The distribution ratio (*D*) was calculated by the ratio between the concentration (radioactivity counts per unit volume) in the organic phase and in the aqueous phase. The separation factors (*SF*) was determined by the ratio of distribution ratios of ^{152,154}Eu (III) to ^{241}Am(III).

Table S3 Distribution ratios and separation factors for the stripping of Eu(III) and Am(III) from **TODGA**-based organic phase into **Phen-2DIBA-**based aqueous phase

Conc. of stripping agents	Eu% in org	Am% in org	$D_{Eu(III)}$	$D_{Am(III)}$	$SF_{Eu/Am}$
1 mM	99.40	98.36	166.90	60.14	2.78
3 mM	98.66	67.40	73.36	2.07	35.44
5 mM	98.09	30.38	51.25	0.44	116.48

Experiment 2: Effect of HNO_3 concentrations on stripping of Eu(III) and Am(III) from **TODGA**-based organic phases.

As it's well known that the extraction abilities of **TODGA** increased with HNO_3 concentrations.[16, 17] Thus dilute HNO_3 solutions were used to back-extracted both Eu(III) and Am(III) from **TODGA**-based organic phases. After separation of the **TODGA** phases as described in the main text and extraction procedures in Page S3, the **TODGA** phases were contacted with different concentrations of dilute HNO_3 solutions, the mixing, phase separation and metal ions concentration quantifications were repeated as in experiment 1, the results were given in Table S4, most of Eu(III) and Am(III) (over 99%) could be back-extracted into aqueous HNO_3 phases from **TODGA**-based organic phases.

Table S4 Distribution ratios for the stripping of Eu(III) and Am(III) from **TODGA**-based organic phase into dilute HNO_3

Conc. of stripping HNO_3	Eu% in org	Am% in org	$D_{Eu(III)}$	$D_{Am(III)}$
0.1 M	1.49	0.44	0.015	0.047
0.05 M	0.79	0.27	0.008	0.030
0.01 M	0.40	0.13	0.004	0.013

Experiment 3: Comparison of extraction performances of **PyTri** and **Phen-2DIBA** under high acidity of 1.5 M HNO_3

PyTri ligand represented one of the most important landmark ligands for hydrophilic Lns(III)/Ans(III) separation.[7] In this experiment, we compared the separation of Eu(III) and Am(III) of the two ligands side by side. Under the same condition as described in the main text, PyTri gave $D_{Eu(III)}$ and $D_{Am(III)}$ of 1.539 and 0.917 with the corresponding $SF_{Eu/Am}$ of 1.68. (Conditions: Organic phase: **TODGA** (0.05 M) in dodecane. Aqueous phase: 5 mM **Phen-2DIBA** or **PyTri** in 1.5 M HNO_3 with 1.5 M $NaNO_3$. O/A=1; Vortex shaker (50 Hz) for 30 minutes at 25±1°C)

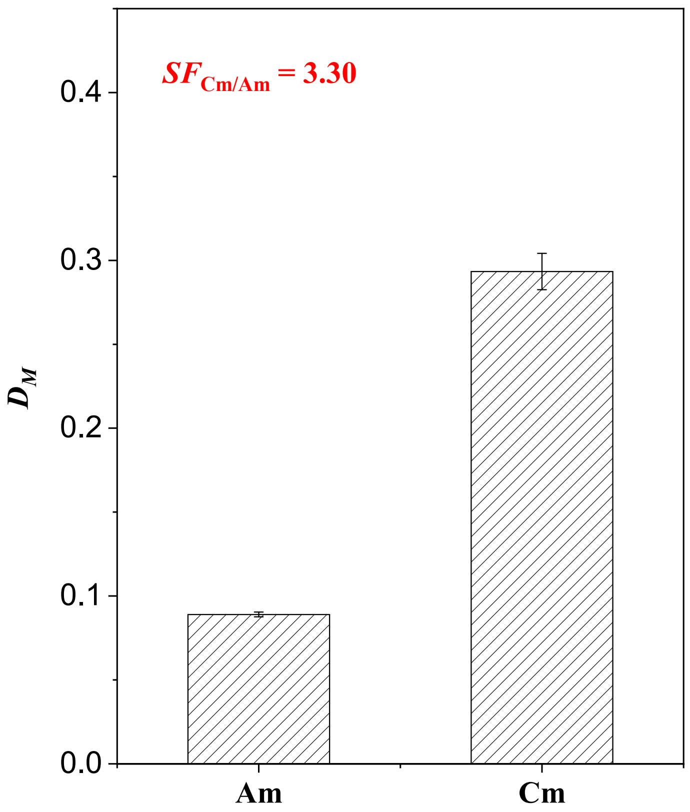

Figure S11. Distribution ratios (D_M) and separation factors (*SF*) obtained in the extraction of ^{241}Am(III) and ^{244}Cm(III) by **TODGA** in the presence of **Phen-2DIBA**. Organic phase: **TODGA** (0.05 M) in dodecane. Aqueous phase: 5 mM **Phen-2DIBA** in 1.5 M HNO_3 with $NaNO_3$. The total concentration of NO_3^- was fixed to 3 M. O/A=1; Vortex shaker (50 Hz) for 30 minutes at 25±1°C.

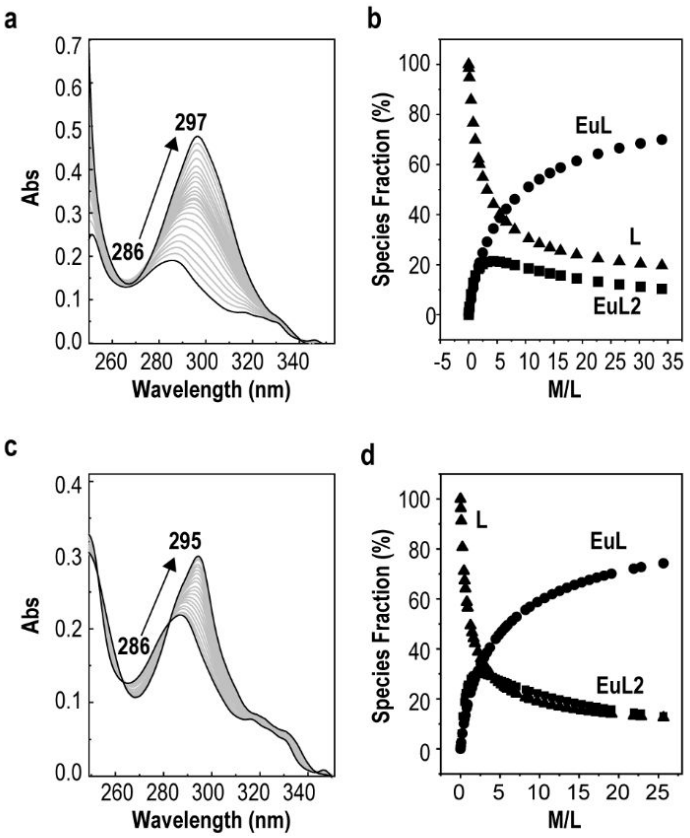

Figure S12. UV-vis absorption spectra titration of **Phen-2DIBA** with $Eu(NO_3)_3$ in 0.01 M HNO_3 (0.1 M NH_4NO_3) (a) and $Eu(ClO_4)_3$ in 0.01 M $HClO_4$ (0.1 M $NaClO_4$) (c). Ligand concentration was 0.01 mM. The 286 and 297 (295 for ClO_4^- system) nm showed the initial and final peak positions at the longer wavelength. (b) and (d) Species distribution of Eu(III) with **Phen-2DIBA** derived from panel (a) and (c).

Table S5 Apparent stability constants for Eu(III) complexes with **Phen-2DIBA** obtained by UV-vis absorption spectra titrations in different ionic medium at 25 ^oC

Ionic medium	Ions	Reactions	Log β
0.01 M $HClO_4$ with 0.1 M $NaClO_4$		$L + Eu^{3+} \rightleftharpoons EuL^{3+}$	4.36±0.03
		$2L + Eu^{3+} \rightleftharpoons EuL_2^{3+}$	10.33±0.52
0.01 M HNO_3 with 0.1 M $NaNO_3$	Eu^{3+}	$L + Eu^{3+} \rightleftharpoons EuL^{3+}$	4.31±0.03
		$2L + Eu^{3+} \rightleftharpoons EuL_2^{3+}$	9.47±0.02
0.01 M HNO_3 with 0.1 M NH_4NO_3		$L + Eu^{3+} \rightleftharpoons EuL^{3+}$	4.36±0.07
		$2L + Eu^{3+} \rightleftharpoons EuL_2^{3+}$	9.49±0.19

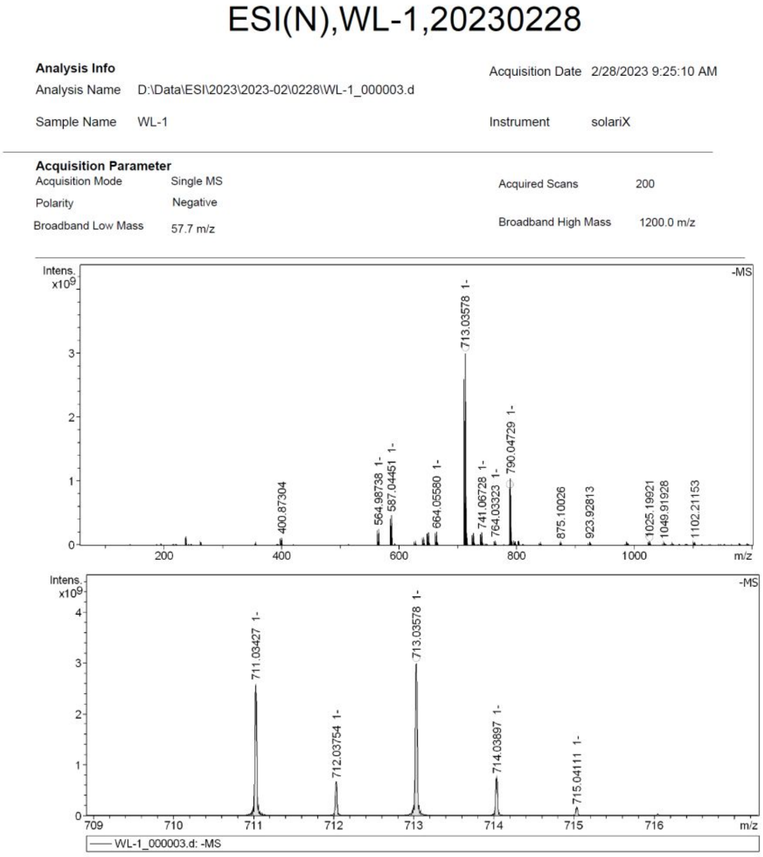

Figure S13. ESI-MS spectrum of **Phen-2DIBA** and $Eu(NO_3)_3{\cdot}6H_2O$ with molecular ratio of 1:1 in methanol.

Table S6 Species analysis of the ESI-MS spectrum (**Phen-2DIBA** and $Eu(NO_3)_3{\cdot}6H_2O$, 1:1 in methanol)

Speculated Structures	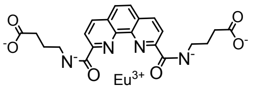	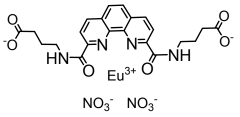	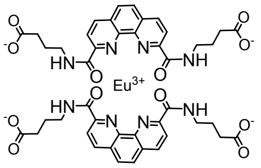
Molecular Formula	$[C_{22}H_{18}EuN_4O_6]^-$	$[C_{22}H_{20}EuN_6O_{12}]^-$	$[C_{44}H_{40}EuN_8O_{12}]^-$
Calculated m/z	587.0444	713.0356	1025.1983
Measured m/z	587.0445	713.0358	1025.1992
Corresponding Species	$[[L-4H]Eu]^-$	$[[L-2H]Eu(NO_3^-)_2]^-$	$[[L-2H]_2Eu]^-$

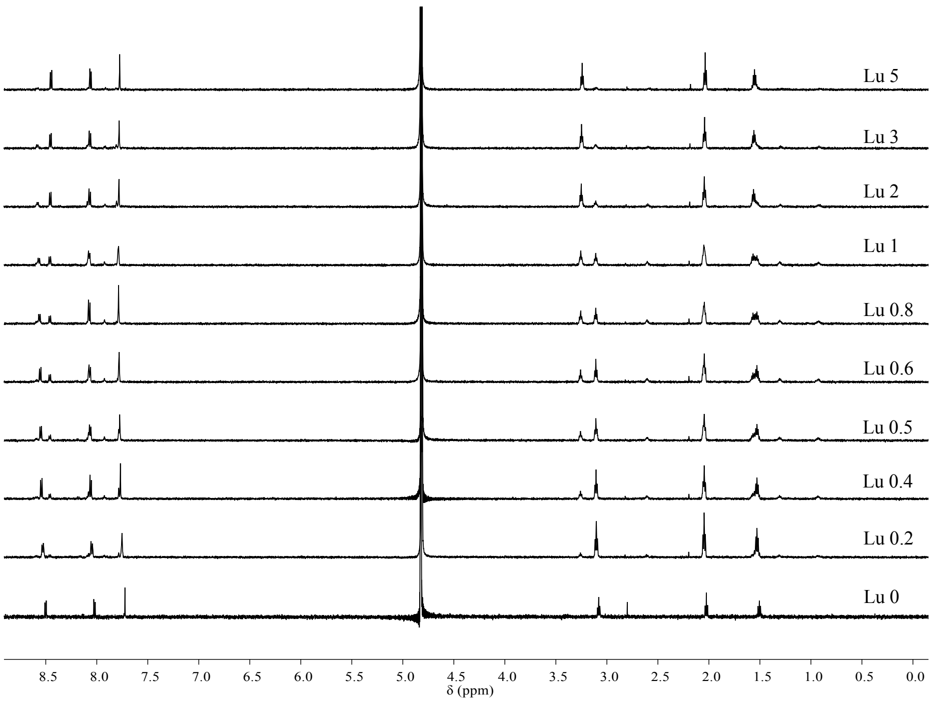

Figure S14. Full NMR spectra for the titration of **Phen-2DIBA** with $Lu(NO_3)_3{\cdot}6H_2O$. Solvent: 1.5 M DNO_3 in D_2O.

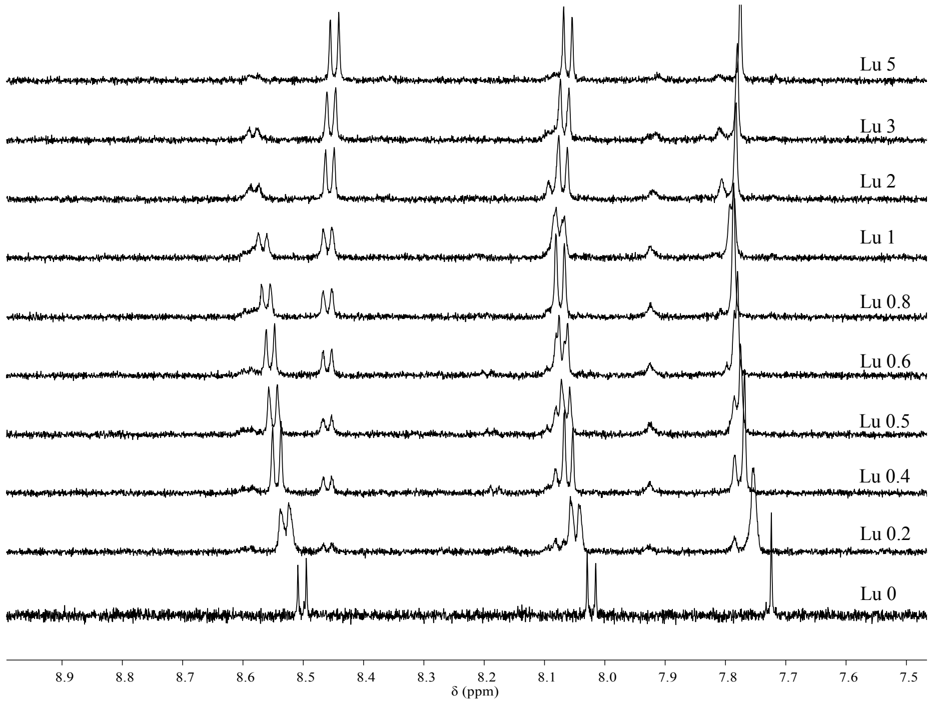

Figure S15. Downfield NMR spectra for the titration of **Phen-2DIBA** with $Lu(NO_3)_3{\cdot}6H_2O$ showing chemical shifts of aromatic parts. Solvent: 1.5 M DNO_3 in D_2O.

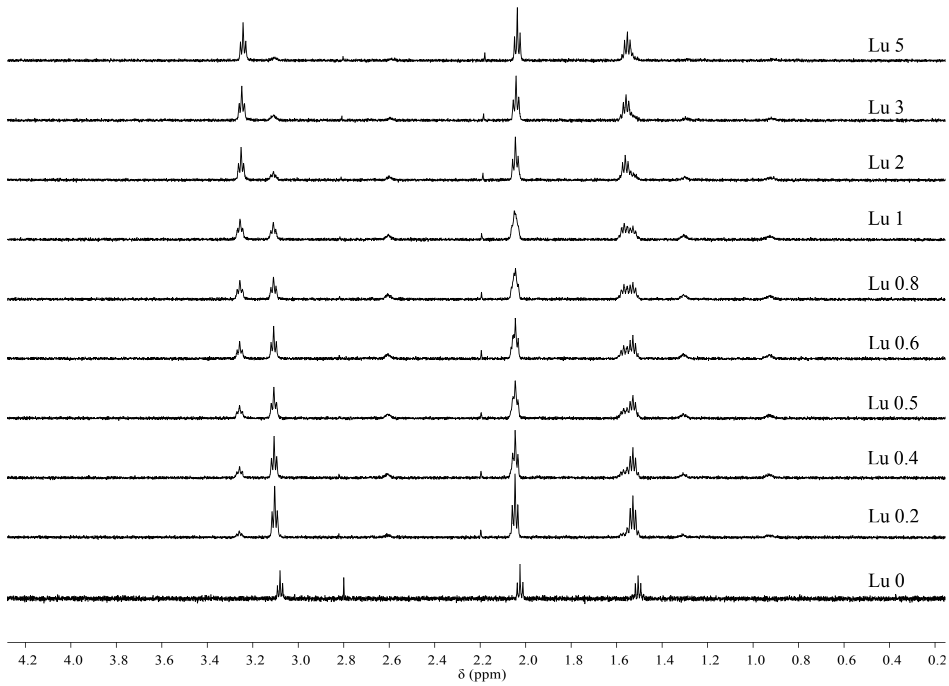

Figure S16. Upfield NMR spectra for the titration of **Phen-2DIBA** with $Lu(NO_3)_3·6H_2O$ showing chemical shifts of alkyl chain parts. Solvent: 1.5 M DNO_3 in D_2O.

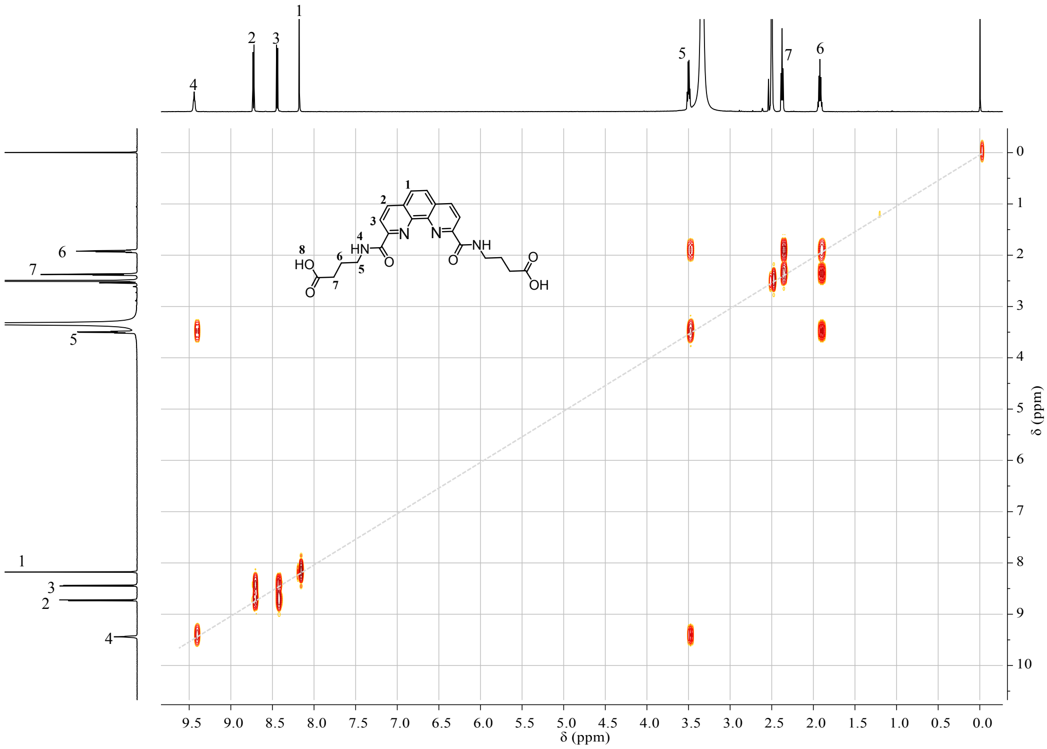

Figure S17. 2D H-H COSY NMR spectra for **Phen-2DIBA** in DMSO-D_6.

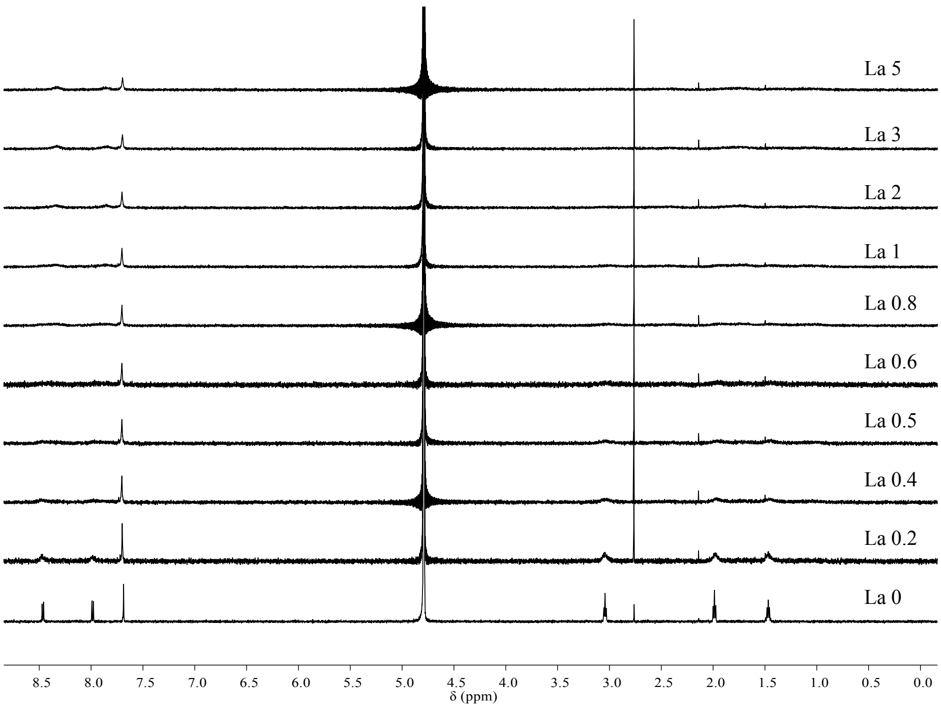

Figure S18. Full NMR spectra for the titration of **Phen-2DIBA** with $La(NO_3)_3 \cdot 6H_2O$ (1/1). Solvent: 1.5 M DNO_3 in D_2O.

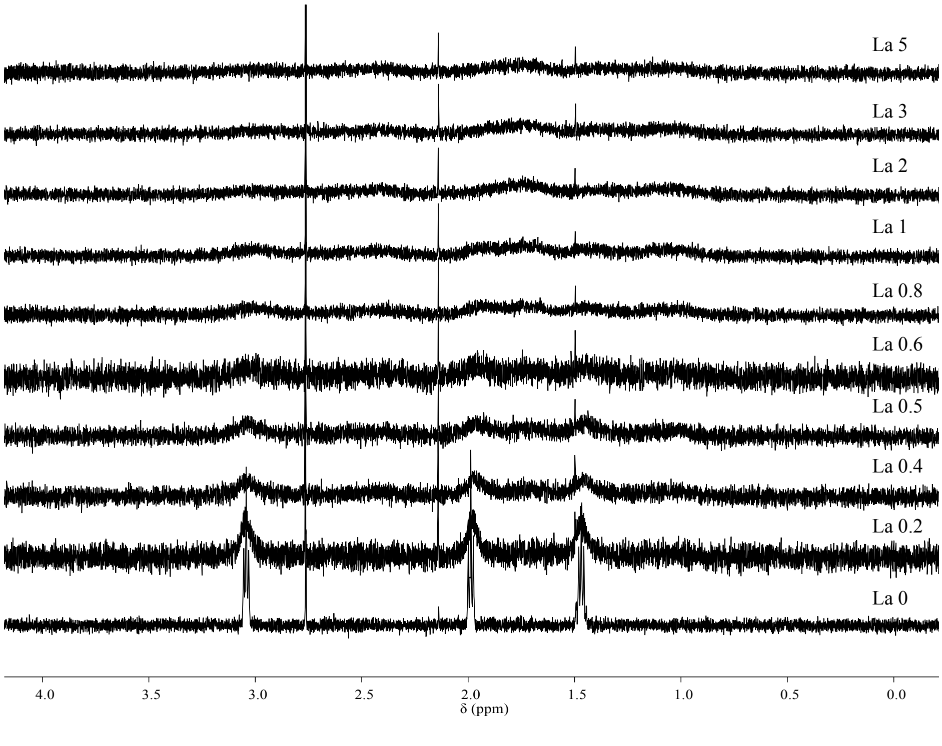

Figure S19. Upfield NMR spectra for the titration of **Phen-2DIBA** with $La(NO_3)_3 \cdot 6H_2O$ (1/1) showing chemical shifts of alkyl chain part. Solvent: 1.5 M DNO_3 in D_2O.

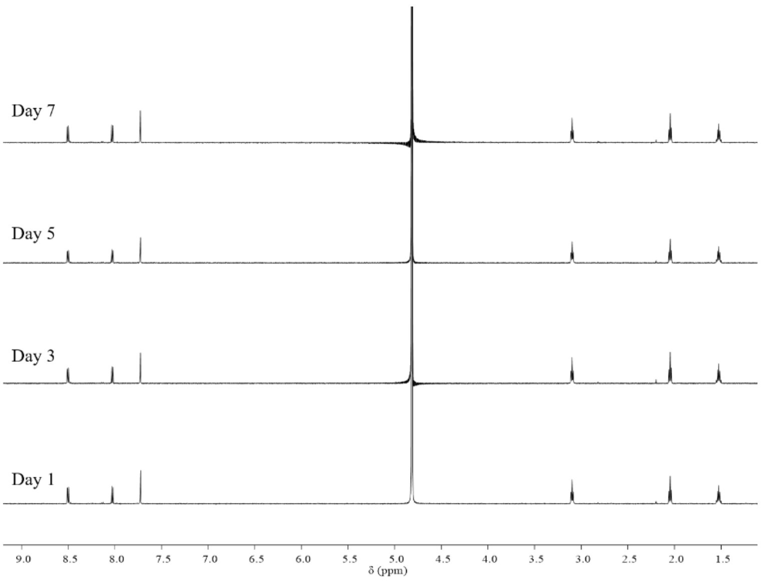

Figure S20. NMR spectra of **Phen-2DIBA** in 1.5 M DNO_3 in D_2O monitored over a week.

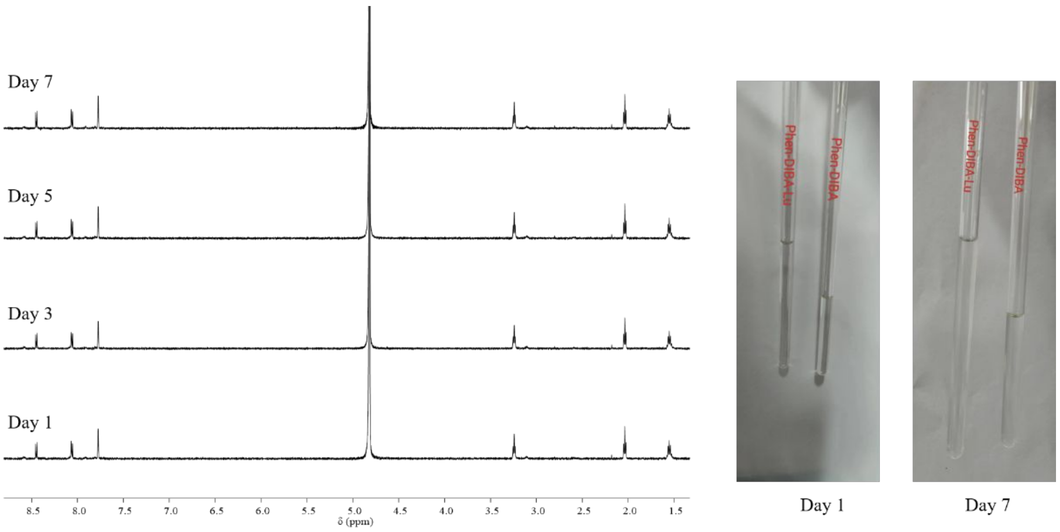

Figure S21. NMR spectra of **Phen-2DIBA** with $Lu(NO_3)_3 \cdot 6H_2O$ (1/1) in 1.5 M DNO_3 in D_2O monitored over a week. The pictures showed the photographs of both ligand and the complexes at different time indicating no solid formed or precipitated out from the solutions.

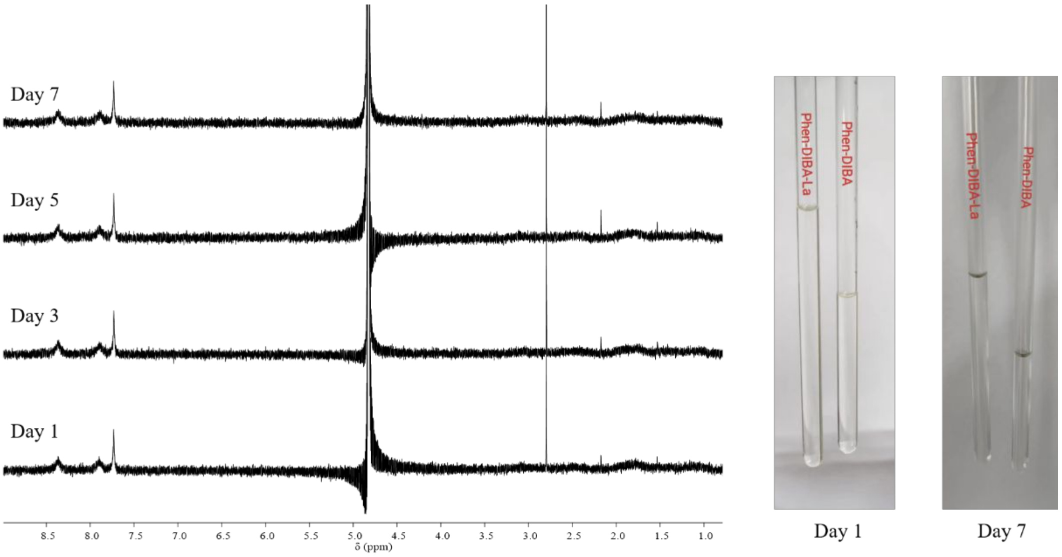

Figure S22. NMR spectra of **Phen-2DIBA** with $La(NO_3)_3{\cdot}6H_2O$ (1/1) in 1.5 M DNO_3 in D_2O monitored over a week. The pictures showed the photographs of both ligand and the complexes at different time indicating no solid formed or precipitated out from the solutions.

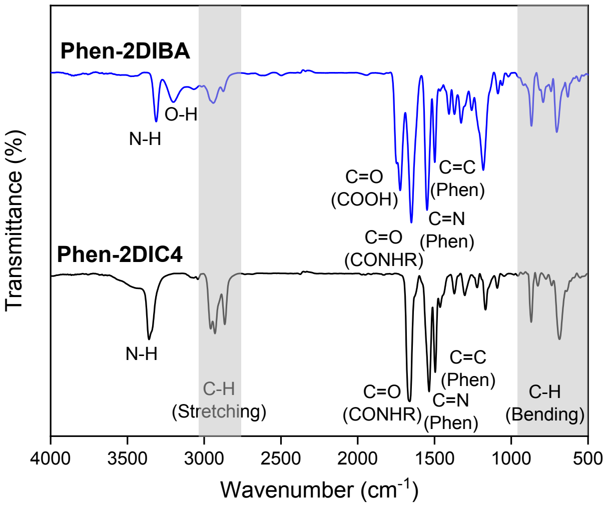

Figure S23. Full IR spectrum of **Phen-2DIC4** and **Phen-2DIBA**.

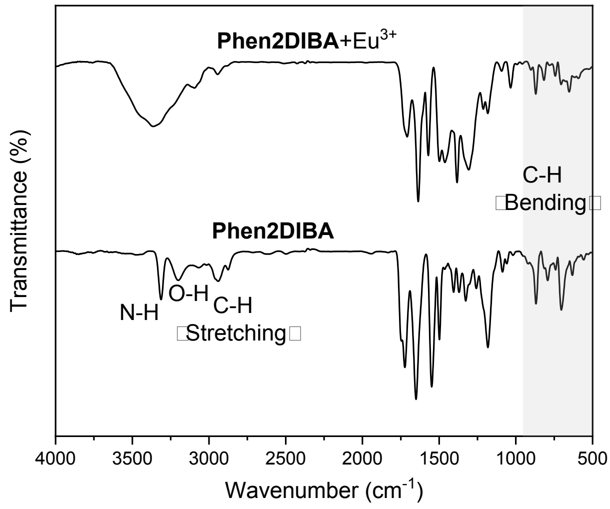

Figure S24. Full IR spectrum of **Phen-2DIBA** and **Phen-2DIBA**/$Eu(NO_3)_3 \cdot 6H_2O$ (1/1).

Supporting Information Note 2.

Single crystals for **Phen-2DIBA**/Eu(III) complexes were grown from 1:1 (v/v) solution of methanol and isopropanol. To ensure the complexation process, 8.8 mg (0.02 mmol) of **Phen-2DIBA** was first suspended in 2 mL methanol, to which 9.0 mg (0.02 mmol) of $Eu(NO_3)_3 \cdot 6H_2O$ in 1 mL methanol was added. The solution turned clear right after the Eu^{3+} was added. The mixture was left stirring at room temperature for 5 hours before evaporated to dryness. Highly red-emissive solid was afforded indicating the formation of the complexes. The as-prepared complexes were redissolved in 2 mL methanol, then 2 mL isopropanol was added carefully. The mixture was left for two days before belt-like crystals were detected at the bottom of the bottle.

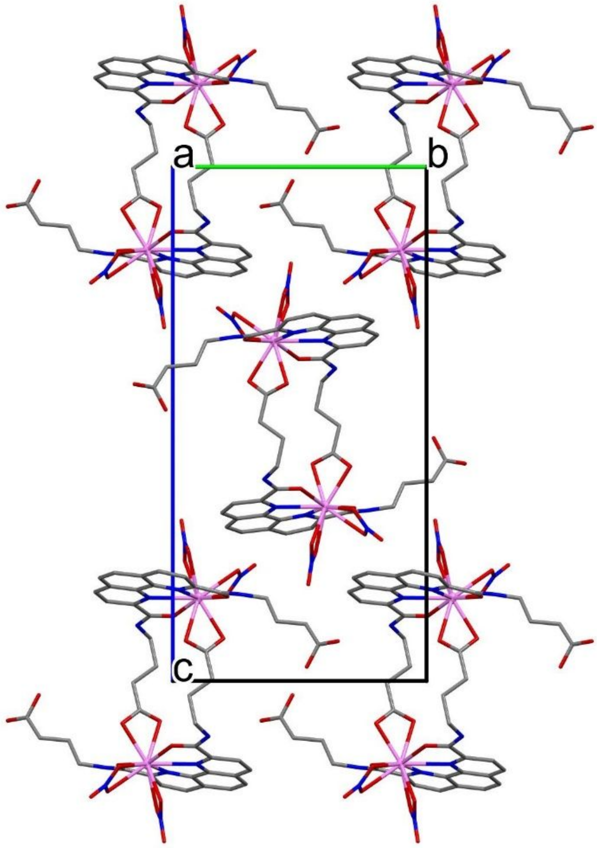

Figure S25. Crystal structure of the unit cell for **Phen-2DIBA**/Eu(III) along a-axis.

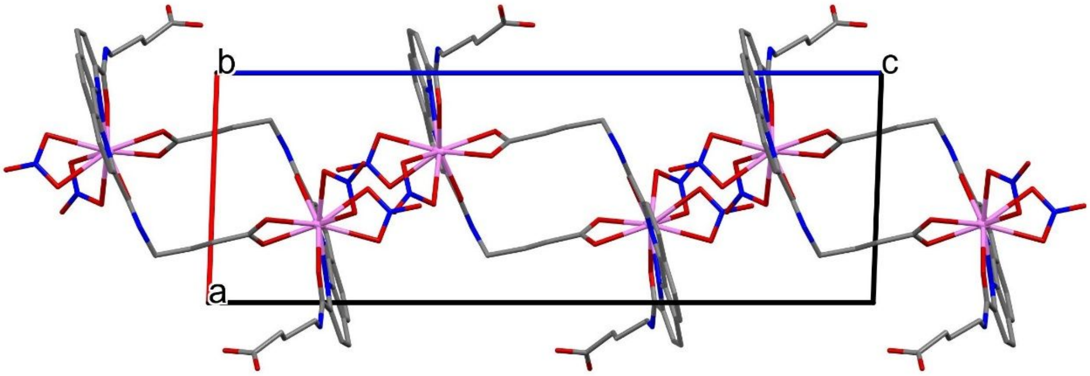

Figure S26. Crystal structure of the unit cell for **Phen-2DIBA**/Eu(III) along b-axis.

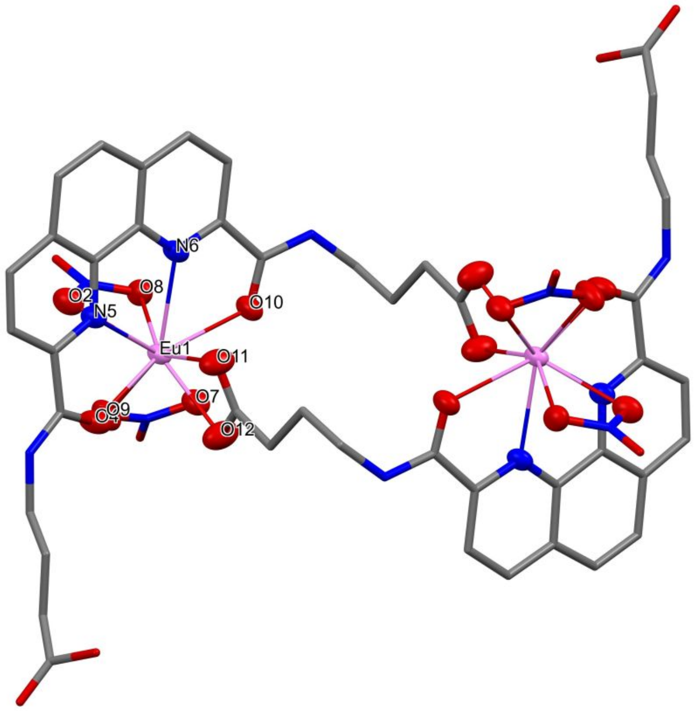

Figure S27. Crystal structure of **Phen-2DIBA**/Eu(III) with coordination atoms labeled around one Eu(III) center.

Table S7 Summary of coordination bond lengths around Eu(III) center

Bond Type / Distance (Å)	Eu-N (phen)	Eu-O (COO^-)	Eu-O (NO_3^-)	Eu-O (imide O)
Eu1-N5	2.605			
Eu1-N6	2.567			
Eu1-O11		2.453		
Eu1-O12		2.452		
Eu1-O2			2.545	
Eu1-O8			2.534	
Eu1-O7			2.584	
Eu1-O9			2.559	
Eu1-O4				2.418
Eu1-O10				2.434

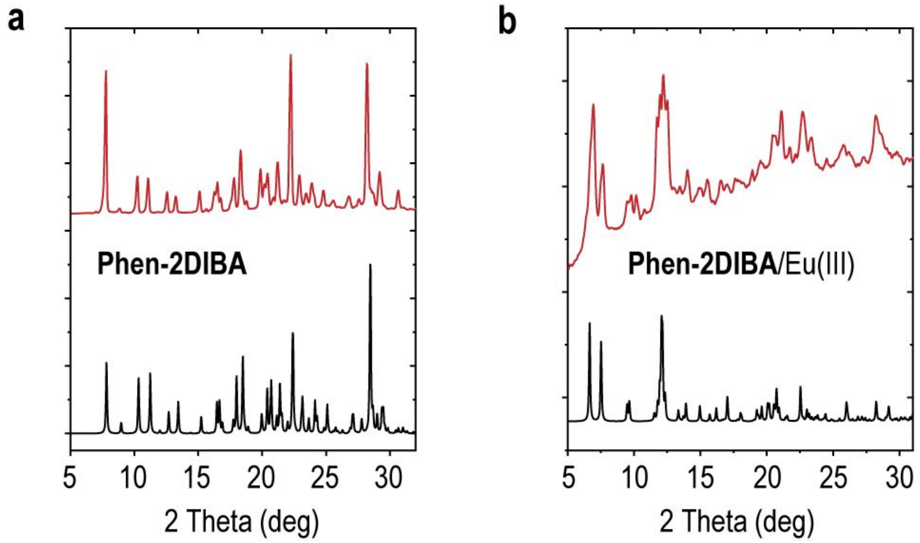

Figure S28. Comparison of PXRD data (red traces) and powder diffraction data derived from single X-ray data (black traces) of **Phen-2DIBA** (a) and **Phen-2DIBA**/Eu(III) (b).

Table S8 Crystal data and structure refinement for **Phen-2DIBA**/Eu(III) complexes

Identification code	**Phen-2DIBA**/Eu(III)
CCDC No.	2248083
Empirical formula	$C_{52}H_{66}Eu_2N_{12}O_{28}$
Formula weight	1611.08
Temperature/K	293(2)
Crystal system	monoclinic
Space group	$P2_1/c$
a/Å	9.16330(10)
b/Å	13.0998(2)
c/Å	26.5452(4)
α/°	90
β/°	92.113(2)
γ/°	90
Volume/Å^3	3184.25(8)
Z	2
ρ_{calc}g/cm^3	1.680
μ/mm^{-1}	14.779
F(000)	1624.0
Crystal size/mm^3	0.3 × 0.08 × 0.08
Radiation	Cu Kα (λ = 1.54184)
2q range for data collection/°	9.49 to 149.068
Index ranges	$-11 \leq h \leq 10$, $-15 \leq k \leq 9$, $-32 \leq l \leq 32$
Reflections collected	18801
Independent reflections	6321 [R_{int} = 0.0765, R_{sigma} = 0.0606]
Data/restraints/parameters	6321/81/441
Goodness-of-fit on F^2	1.078
Final R indexes [I>=2σ (I)]	R_1 = 0.0633, wR_2 = 0.1733
Final R indexes [all data]	R_1 = 0.0710, wR_2 = 0.1784
Largest diff. peak/hole / e Å^{-3}	1.98/-1.96

Table S9 Crystal data and structure refinement for **Phen-2DIBA**

Identification code	**Phen-2DIBA**
CCDC No.	2249573
Empirical formula	$C_{22}H_{22}N_4O_6$
Formula weight	438.43
Temperature/K	180.00(10)
Crystal system	monoclinic
Space group	$P2_1/n$
a/Å	13.4257(4)
b/Å	8.8527(3)
c/Å	17.2813(5)
α/°	90
β/°	98.062(3)
γ/°	90
Volume/Å^3	2033.65(11)
Z	4
ρ_{calc}g/cm^3	1.432
μ/mm^{-1}	0.106
F(000)	920.0
Crystal size/mm^3	0.16 × 0.15 × 0.03
Radiation	Mo Kα (λ = 0.71073)
2q range for data collection/°	5.18 to 58.902
Index ranges	$-18 \le h \le 14$, $-12 \le k \le 11$, $-23 \le l \le 23$
Reflections collected	14557
Independent reflections	5117 [R_{int} = 0.0268, R_{sigma} = 0.0348]
Data/restraints/parameters	5117/0/291
Goodness-of-fit on F^2	1.046
Final R indexes [I>=2σ (I)]	R_1 = 0.0407, wR_2 = 0.1043
Final R indexes [all data]	R_1 = 0.0559, wR_2 = 0.1109
Largest diff. peak/hole / e Å^{-3}	0.29/-0.30

Supporting Information Note 3.

The geometry optimizations of complexes were performed by the density functional theory (DFT) and B3LYP functional using Gaussain 09.[18, 19] The 6-311G(d) basis set was applied for the elements in the first three periods (C, H, O, N). The quasi-relativistic small-core pseudopotential ECP28MWB along with the corresponding ECP28MWB_SEG segment basis set were used to describe Eu(III) and Nd(III) and ECP60MWB_SEG segment basis set were used to describe Am(III).[20, 21] Generally, the accuracy of the calculation results of small nuclear pseudopotential is better than that of large nuclear pseudopotential. Based on the results of this work, the 1:1 type composition [$M(NO_3)_3L$] was used to describe the structure of Eu, Nd and Am. Since this work only explore the differences in the properties of complexes formed by different metal ions, we have used a simplified complex structure to shorten the calculation time. As shown in Table S10, the average Eu-N bond length was longer than that of Am-N indicating the softer nature of Ans(III) than Lns(III). While the relative shorter Eu-O bonds showed the preferable binding of Lns(III) to harder O, this agreed well with the reported data for other hydrophilic ligands.[6]

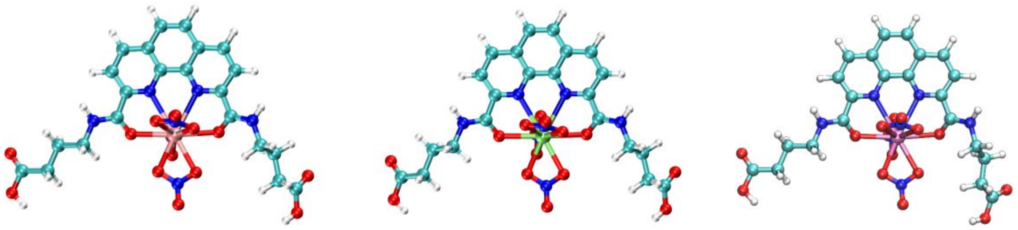

Figure S29. Optimized structures of the 1:1 complexes of **Phen-2DIBA** with Eu(III) (left), Am(III) (middle) and Nd(III) (right) with the simplified architecture of [$M(NO_3)_3L$].

Table S10 Calculated bond length (Å) and bond types of **Phen-2DIBA** with Eu(III), Am(III) and Nd(III).

Metal Complexes	M-N1	M-N2	M-O1	M-O2
$Eu(NO_3)_3L$	2.829	2.792	2.484	2.491
$Am(NO_3)_3L$	2.785	2.755	2.510	2.528
$Nd(NO_3)_3L$	2.825	2.836	2.536	2.528

Note: N1, N2, O1 and O2 refer to the binding sites N and O with clockwise numbering

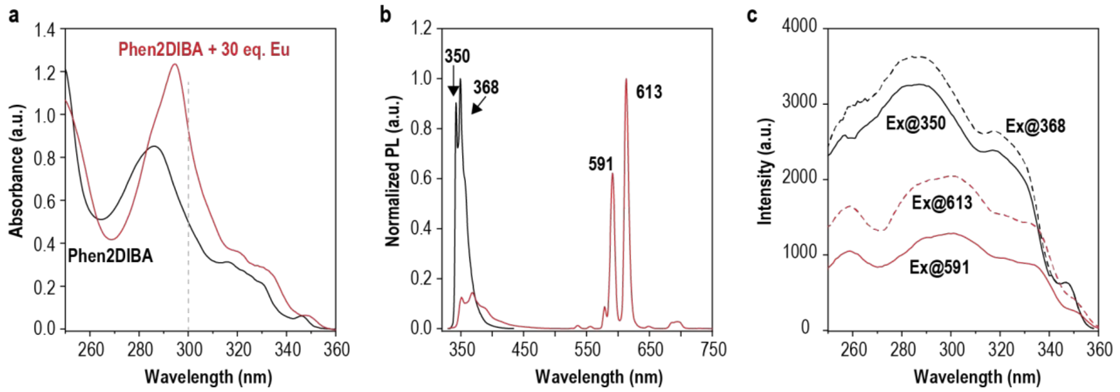

Figure S30. Absorption (a), emission (b) and excitation (c) spectra of **Phen-2DIBA** (black) and with 30 eq. $Eu(NO_3)_3 \cdot 6H_2O$ (red). Conditions: 5 mM of **Phen-2DIBA** in 0.01 M HNO_3, the excitation wavelengths of PL in panel (b) were 300 nm as indicated in dash line in panel (a). The slits for PL and excitation spectra were 5/5.

Supporting Information Note 4.

It's been known that the fluorescence lifetimes of Eu(III) complexes were closely related to the number of water molecules in the first coordination sphere of the metal center (OH vibration quenched the emission from the metal).[22] An empirical equation was proposed by Barthelemy and Choppin to show the observed decay constant (the reciprocal fluorescence lifetime, unit in ms^{-1}) was linearly corelated to the coordination water molecules (n_{H2O}) following $n_{H2O} = 1.05\ K_{H2O} - 0.70$ with an inherent uncertainty of ± 0.5 water molecules.[23] Thus, the number of water molecules in the first coordination sphere of Eu(III) ions during the titration were monitored indirectly by calculation of the emission decay lifetimes after each titration. Perchlorate salts were used to eliminate the influence of counterions. In all our experiments, biexponential fittings were observed except the initial $Eu(ClO_4)_3$ solution. All decays curves and the first/last data with fitting cures were given in Figure S31, the fitting equation and the fitted results were depicted in Table S11.

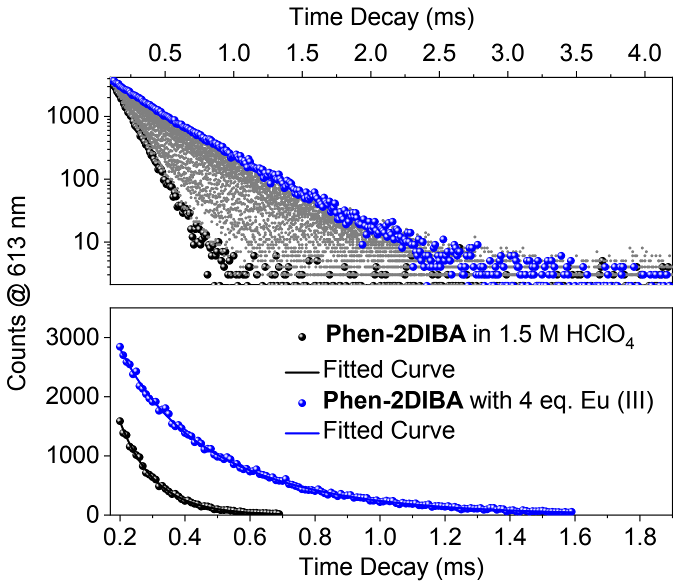

Figure S31. Emission decays as a function of time deduced from TRLFS titration experiments shown in Figure 4c in the main text with $Eu(ClO_4)_3$ and **Phen-2DIBA**/$Eu(ClO_4)_3$ ratio of 1:4 data emphasized in black and blue, respectively (top). Representative lifetime fitting curves (bottom). Conditions: $C_{Ligand}/C_{Eu(III)}$ = 4 mM/1 mM, titrant: **Phen-2DIBA** in 1.5M $HClO_4$, I=1M $HClO_4$, 1.585 mL of titrant was added. V_0=1.6 ml.

Table S11 Lifetime fitting results

	Solution components	Fitted data
Entry 1	1 mM $Eu(ClO_4)_3$ in 1.5M $HClO_4$, I=1M $NaClO_4$,	t_1=108.57, B_1=4372.76, χ^2=0.984
Entry 27	4 equivalent of **Phen-2DIBA** to $Eu(ClO_4)_3$ in 1.5M $HClO_4$, I=1M $NaClO_4$,	t_1=168.51, B_1=1407.43 ; t_2=363.85, B_2=2694.25 ; χ^2=0.989

Fitted equation: $R(t) = B1\exp\left(-\frac{t}{\tau1}\right) + B2\exp\left(-\frac{t}{\tau2}\right) + B3\exp\left(-\frac{t}{\tau3}\right)$

SI References

1. Duan, L.; Fan, J.; Tian, D.; Yan, Q.; Zhang, X.; Li, P.; Xu, C.; Wang, L., A novel and versatile precursor for the synthesis of highly preorganized tetradentate ligands based on phenanthroline and their binding properties towards lanthanides(III) ions. *Colloid Surf. A-Physicochem. Eng. Asp.* **2022,** *647*, 129089.

2. Alderighi, L.; Gans, P.; Ienco, A.; Peters, D.; Sabatini, A.; Vacca, A., Hyperquad simulation and speciation (HySS): a utility program for the investigation of equilibria involving soluble and partially soluble species. *Coord. Chem. Rev.* **1999,** *184* (1), 311-318.

3. Geist, A.; Müllich, U.; Magnusson, D.; Kaden, P.; Modolo, G.; Wilden, A.; Zevaco, T., Actinide(III)/Lanthanide(III) Separation Via Selective Aqueous Complexation of Actinides(III) using a Hydrophilic 2,6-Bis(1,2,4-Triazin-3-Yl)-Pyridine in Nitric Acid. *Solvent Extr. Ion Exch.* **2012,** *30* (5), 433-444.

4. Lewis, F. W.; Harwood, L. M.; Hudson, M. J.; Geist, A.; Kozhevnikov, V. N.; Distler, P.; John, J., Hydrophilic sulfonated bis-1,2,4-triazine ligands are highly effective reagents for separating actinides(iii) from lanthanides(iii) via selective formation of aqueous actinide complexes. *Chem. Sci.* **2015,** *6* (8), 4812-4821.

5. Wilden, A.; Modolo, G.; Kaufholz, P.; Sadowski, F.; Lange, S.; Sypula, M.; Magnusson, D.; Müllich, U.; Geist, A.; Bosbach, D., Laboratory-Scale Counter-Current Centrifugal Contactor Demonstration of an Innovative-SANEX Process Using a Water Soluble BTP. *Solvent Extr. Ion Exch.* **2015,** *33* (2), 91-108.

6. Ren, P.; Huang, P.-w.; Yang, X.-f.; Zou, Y.; Tao, W.-q.; Yang, S.-l.; Liu, Y.-h.; Wu, Q.-y.; Yuan, L.-y.; Chai, Z.-f.; Shi, W.-q., Hydrophilic Sulfonated 2,9-Diamide-1,10-phenanthroline Endowed with a Highly Effective Ligand for Separation of Americium(III) from Europium(III): Extraction, Spectroscopy, and Density Functional Theory Calculations. *Inorg. Chem.* **2021,** *60* (1), 357-365.

7. Macerata, E.; Mossini, E.; Scaravaggi, S.; Mariani, M.; Mele, A.; Panzeri, W.; Boubals, N.; Berthon, L.; Charbonnel, M.-C.; Sansone, F.; Arduini, A.; Casnati, A., Hydrophilic Clicked 2,6-Bis-triazolyl-pyridines Endowed with High Actinide Selectivity and Radiochemical Stability: Toward a Closed Nuclear Fuel Cycle. *J. Am. Chem. Soc.* **2016,** *138* (23), 7232-7235.

8. Weßling, P.; Maag, M.; Baruth, G.; Sittel, T.; Sauerwein, F. S.; Wilden, A.; Modolo, G.; Geist, A.; Panak, P. J., Complexation and Extraction Studies of Trivalent Actinides and Lanthanides with Water-Soluble and CHON-Compatible Ligands for the Selective Extraction of Americium. *Inorg. Chem.* **2022,** *61* (44), 17719-17729.

9. Edwards, A. C.; Mocilac, P.; Geist, A.; Harwood, L. M.; Sharrad, C. A.; Burton, N. A.; Whitehead, R. C.; Denecke, M. A., Hydrophilic 2,9-bis-triazolyl-1,10-phenanthroline ligands enable selective Am(iii) separation: a step further towards sustainable nuclear energy. *Chem. Commun.* **2017,** *53* (36), 5001-5004.

10. Ruff, C. M.; Müllich, U.; Geist, A.; Panak, P. J., Complexation of Cm(iii) and Eu(iii) with a hydrophilic 2,6-bis(1,2,4-triazin-3-yl)-pyridine studied by time-resolved laser fluorescence spectroscopy. *Dalton Trans.* **2012,** *41* (48), 14594-14602.

11. Jensen, M. P.; Chiarizia, R.; Shkrob, I. A.; Ulicki, J. S.; Spindler, B. D.; Murphy, D. J.; Hossain, M.; Roca-Sabio, A.; Platas-Iglesias, C.; de Blas, A.; Rodríguez-Blas, T., Aqueous Complexes for Efficient Size-based Separation of Americium from Curium. *Inorg. Chem.* **2014,** *53* (12), 6003-6012.

12. Kaufholz, P.; Modolo, G.; Wilden, A.; Sadowski, F.; Bosbach, D.; Wagner, C.; Geist, A.; Panak, P. J.; Lewis, F. W.; Harwood, L. M., Solvent Extraction and Fluorescence Spectroscopic Investigation of the Selective Am(III) Complexation with TS-BTPhen. *Solvent Extr. Ion Exch.* **2016,** *34* (2), 126-140.

13. Wagner, C.; Müllich, U.; Geist, A.; Panak, P. J., Selective Extraction of Am(III) from PUREX Raffinate: The AmSel System. *Solvent Extr. Ion Exch.* **2016,** *34* (2), 103-113.

14. Bhattacharyya, A.; Gadly, T.; Kanekar, A. S.; Ghosh, S. K.; Kumar, M.; Mohapatra, P. K., First Report on the Separation of Trivalent Lanthanides from Trivalent Actinides Using an Aqueous Soluble Multiple N-Donor Ligand, 2,6-bis(1H-tetrazol-5-yl)pyridine: Extraction, Spectroscopic, Structural, and Computational Studies. *Inorg. Chem.* **2018,** *57* (9), 5096-5107.

15. Scaravaggi, S.; Macerata, E.; Galletta, M.; Mossini, E.; Casnati, A.; Anselmi, M.; Sansone, F.; Mariani, M., Hydrophilic 1,10-phenanthroline derivatives for selective Am(III) stripping into aqueous solutions. *J. Radioanal. Nucl. Chem.* **2015,** *303* (3), 1811-1820.

16. Ansari, S. A.; Pathak, P. N.; Manchanda, V. K.; Husain, M.; Prasad, A. K.; Parmar, V. S., N,N,N′,N′-Tetraoctyl Diglycolamide (TODGA): A Promising Extractant for Actinide-Partitioning from High-Level Waste (HLW). *Solvent Extr. Ion Exch.* **2005,** *23* (4), 463-479.

17. Magnusson, D.; Christiansen, B.; Glatz, J. P.; Malmbeck, R.; Modolo, G.; Serrano-Purroy, D.; Sorel, C., Demonstration of a TODGA based Extraction Process for the Partitioning of Minor Actinides from a PUREX Raffinate. *Solvent Extr. Ion Exch.* **2009,** *27* (1), 26-35.

18. M. Frisch, G. Trucks, H. B. Schlegel, G. Scuseria, M. Robb, J. Cheeseman, G. Scalmani, V. Barone, B. Mennucci and G. Petersson, Gaussian 09, Revision A.1, Gaussian, Inc., Wallingford, CT, 2009, vol. 200.

19. Lee, C.; Yang, W.; Parr, R. G., Development of the Colle-Salvetti correlation-energy formula into a functional of the electron density. *Phys. Rev. B* **1988,** *37* (2), 785-789.

20. Cao, X.; Dolg, M., Segmented contraction scheme for small-core lanthanide pseudopotential basis sets. *J. Mol. Struc-THEOCHM.* **2002,** *581* (1), 139-147.

21. Cao, X.; Dolg, M., Segmented contraction scheme for small-core actinide pseudopotential basis sets. *J. Mol. Struc-THEOCHM.* **2004,** *673* (1), 203-209.

22. Horrocks, W. D., Jr.; Sudnick, D. R., Lanthanide ion probes of structure in biology. Laser-induced luminescence decay constants provide a direct measure of the number of metal-coordinated water molecules. *J. Am. Chem. Soc.* **1979,** *101* (2), 334-340.

23. Barthelemy, P. P.; Choppin, G. R., Luminescence study of complexation of europium and dicarboxylic acids. *Inorg. Chem.* **1989,** *28* (17), 3354-3357.
