# Supplementary material for: A Simple yet Efficient Hydrophilic Phenanthroline-Based Ligand for Selective Am(III) Separation under High Acidity
Source: ACS Cent Sci. 2023 Jul 14;9(8):1642–9. doi: 10.1021/acscentsci.3c00504 (PMC10451031; doi:10.1021/acscentsci.3c00504)
Supplement: Supplementary file 2 — oc3c00504_si_002.pdf [file oc3c00504_si_002.pdf]

oc-2023-00504e.R1

Name: Peer Review Information for "A Simple yet Efficient Hydrophilic Phenanthroline-Based Ligand for Selective Am(III) Separation under High Acidity"

First Round of Reviewer Comments

Reviewer: 1

Comments to the Author

Comment

The draft entitled " A Simple yet Efficient Hydrophilic Phenanthroline-Based Ligand for Selective Am (III) Separation under High Acidity " by Deshun Tian has reported a simple yet efficient carboxylic-group modified hydrophilic phenanthroline-based tetradentate N, O-ligand which displayed satisfactory Eu(III)/Am(III) and Cm(III)/Am(III) separation capabilities under high acidity. The solvent extraction behaviour and mechanism is well characterized by UV-vis absorption, NMR titrations, ESI-MS, IR and single crystal structure. I'd like to recommend publication in ACS Central Science after the following concerns have been fully addressed.

1、 In figure 1a, the synthesis procedure is not complete, and Phen-2DIBA is the product of acidification. The ligand has poor water solubility but can dissolve in a 1.5 M acidic solution. Explain the reason. Is the ligand stable under high acidity?

2、 Figure S5 is not clear, and the current version cannot clearly display the structural information of hydrogen bonds. In addition, the authors should add a table indicating the bond length and angle information of hydrogen bonds.

3、 In page 7, $NaNO_3$ was used to control the ion strength in order to investigate the effect of nitrate ion on the extraction process. In page 9, Et_4NNO_3 was used to control the ion strength in order to get further information about the complexation behaviors of Phen-2DIBA with the trivalent metal ions in solution. Why did the authors use different nitrates to control the ion strength and whether different cations would affect the results?

4、 Page 10, the understanding of 'red-shifted' and 'blue-shifted' is incorrected.

5、 Page 10 and page 11, 'The 10-coordinated architectures were common for Lns(III)/Ans(III) complexes while, to the best of our knowledge, this was the first report on this kind of dimer-like two-metal-two-ligand coordination mode for Eu(III) complexes.' Binuclear rare earth complexes are very common. Phen-2DIBA-Eu2 is not the first Eu(III) complex with 'dimer-like two-metal-two-ligand coordination mode'. Several Eu(III) complexes (CCDC NO. 1059019, 1953922, 832850…) exhibit similar coordination mode.

6、 PXRD of complex Phen-2DIBA-Eu2 should be provided to confirm purity. Cif and checkcif documents should also be provided.

7、 The authors need to figure out the effect of different concentrations of salt agents on the masking effect.

8、 The authors need to provide the relevant back-extraction experiments date. The authors need to clarify the effect of different masking agent concentrations on the back-extraction effect and make a relevant analysis of the back-extraction mechanism.

9、 The hydrophilic ligand only enhances the separation factor of TODGA appropriately, so please pay attention to the wording of the paper.

Reviewer: 2

Comments to the Author

This work is interesting and can be potentially published in ACS Central Science after following suggested revisions.

1. I can see the ligand design philosophy is similar to the previously proposed “hard/soft” combined strategy, where relatively soft N donor in phenanthroline and hard O donor in carboxylate are both utilized for Ln/Am coordination. The authors achieve record high separation factors of Eu/Am and Am/Cm in highly acidic condition by a masking effect in aqueous solution with such a hydrophilic ligand. A question needs to be considered is that does such a high separation capability originate majorly from the special ligand design or just from the unique masking method apart from the traditional solvent extraction. For instance, can author try a previously reported hydrophilic “hard/soft” ligand and try the masking separation to see the capability difference? This may provide a clue.

2. Another critical data authors may seriously consider to obtain (if authors have such a capability ideally, or at least DFT calculation with a model assuming Am/Eu structures are isotypic) is to grow the single crystal of Am complexes with the new ligand, determine the crystal structure, and compare the Am-N, Am-O distances to Eu-N, Eu-O (maybe Nd-N, Nd-O is better because Am3+ has nearly identical ionic radius with Nd3+, not Eu3+), and then compare these data with other well studied ligand systems. This would tell much better whether the ligand design amplifies the 4f/5f bonding difference and the advtantages.

3. There are several important progresses in Am/Ln separation that are missed in the reference list.

Author's Response to Peer Review Comments:

We would like to thank all the editors for giving us the opportunity to revised the current manuscript entitled “A Simple yet Efficient Hydrophilic Phenanthroline-Based Ligand for Selective Am(III) Separation under High Acidity” and the precious time for all the reviewers to evaluate the work. Points to address both the editors and reviewers comments could be found in the attached files.

Formatting Needs:

1. AU LIST ORDER: Please ensure the order of author names matches exactly between the manuscript file and in Paragon Plus.

   Response: The order of all the author names were double-checked to ensure the consistence between the main text, supporting information and the Paragon Plus system.

2. TOC GRAPHIC: Please move the TOC graphic (and Synopsis) to the last page of the manuscript, after the References.

   Response: The TOC graphic together with the Synopsis were moved accordingly.

3. SYNOPSIS: Please label the Synopsis.

   Response: The Synopsis was labeled and moved back to the end of the main text.

4. FIG. 4: Figure 4 includes a reference notation. Please confirm that this pertains only to data, and not the figure itself. If any of your graphics have been previously published, this should be stated in the caption in the following format: "Reproduced from [COMPLETE REFERENCE CITATION]. Copyright [YEAR] Publisher Name", and copyright permission should be obtained from any non-ACS publications. Upload the copyright permissions as "Other files for editors only" with your revision submission. For details, see http://pubs.acs.org/page/copyright/permissions_otherpub.html.

   Response: The reference cited in Figure 4 caption was only used to clarify the origin of the 1383 cm^{-1} peak (from NO_3^-) and has no relation to the figure itself. To avoid misleading, the original wording was changed by deleting and moving the explanation of "the peak at around 1380 cm^{-1} was from nitrate ion[43]" from Figure 4 caption to the main text (Page 9).

5. SI STATEMENT: Because your manuscript is accompanied by Supporting Information for publication, a brief description of the supplementary material is required in the manuscript. The appropriate format is: Supporting Information (header), followed by a brief statement in nonsentence format listing the contents of the material supplied as Supporting Information.

   Response: Brief description of the Supporting Information was added in the main text before the References sections.

6. SI PAGINATION: SI pages must be numbered consecutively, starting with page S1.

   Response: SI pages have been numbered accordingly.

7. SI REFS: Please add titles to the references in your Supporting Information file.

   Response: The references in the Supporting Information file were reformed to ACS format.

Reviewer: 1
Recommendation: Publish in ACS Central Science after minor revisions noted.

Comments: Review attached. The draft entitled " A Simple yet Efficient Hydrophilic Phenanthroline-Based Ligand for Selective Am (III) Separation under High Acidity " by Deshun Tian has reported a simple yet efficient carboxylic-group modified hydrophilic phenanthroline-based tetradentate N, O-ligand which displayed satisfactory Eu(III)/Am(III) and Cm(III)/Am(III) separation capabilities under high acidity. The solvent extraction behavior and mechanism is well characterized by UV-vis absorption, NMR titrations, ESI-MS, IR and single crystal structure. I'd like to recommend publication in ACS Central Science after the following concerns have been fully addressed.

Response: Thanks for reviewing the current manuscript and the positive comments given. We have carefully addressed all the concerns accordingly in a point-to-point manner as given below:

1. In figure 1a, the synthesis procedure is not complete, and Phen-2DIBA is the product of acidification. The ligand has poor water solubility but can dissolve in a 1.5 M acidic solution. Explain the reason. Is the ligand stable under high acidity?

   Response 1: The synthesis of the ligand **Phen-2DIBA** was actually reported in our previous publication (*Colloid Surf. A-Physicochem. Eng. Asp.* **2022**, *647*, 129089) and we have cross-referred the synthesis of the ligand in the main text (Ref 38).

   The ligand itself displayed poor water solubility while was soluble in HNO_3 solution with concentration higher than 1.25 M. This phenomenon was quite common in literature and was attributed to the protonation of the polypyridyl backbone (*Chem. Rev.* **2013**, *113*, 1199-1236). To clarify this point, we have added "probably because of the protonation of the phenanthroline backbone" in the main text after the description of the ligand solubility (Page 5).

   The ligand stability under high acidity is important for discussion the extraction capability of the hydrophilic ligand. Thanks for pointing this out. To answer this question, we have monitored the NMR spectra of the ligand, La(III) complexes and Lu(III) complexes during a period of a week. The HNO_3 concentrations were set to 1.5 M as the ligand showed the best separation at this acidity. La(III) and Lu(III) cations were chosen because they represented the largest and smallest cations among all Lns(III) and their diamagnetic nature. The results were given in the following figures:

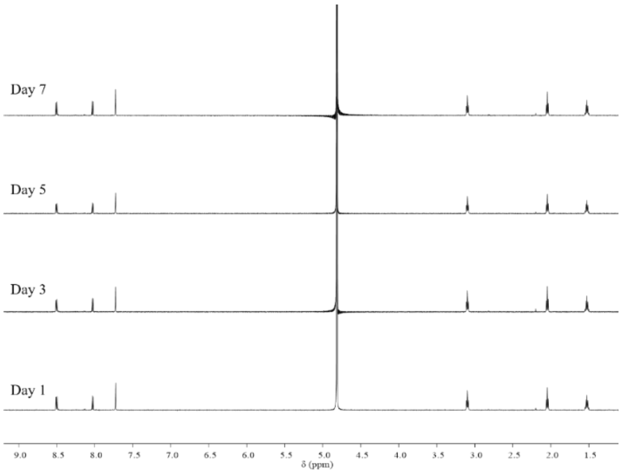

Figure R1. NMR spectra of **Phen-2DIBA** in 1.5 M DNO_3 in D_2O monitored over a week.

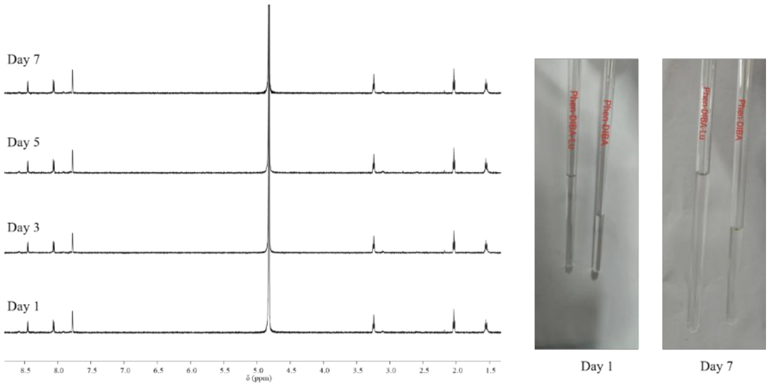

Figure R2. NMR spectra of **Phen-2DIBA** with $Lu(NO_3)_3{\cdot}6H_2O$ (1/1) in 1.5 M DNO_3 in D_2O monitored over a week. The pictures showed the photographs of both ligand and the complexes at different time scales indicating no solid formed o precipitated out from the solutions.

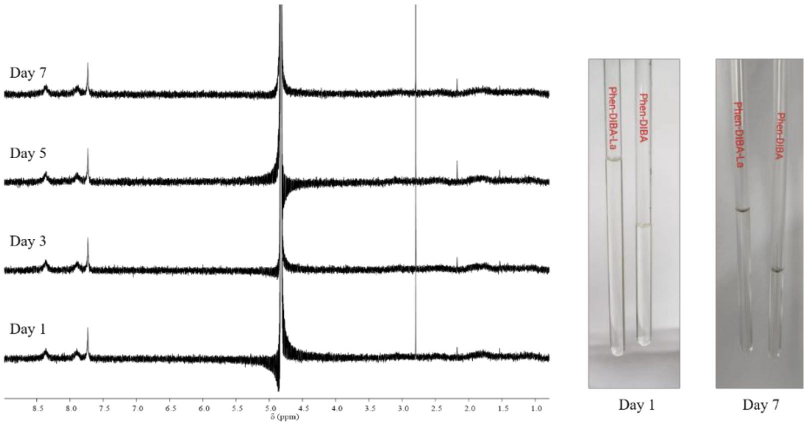

Figure R3. NMR spectra of **Phen-2DIBA** with $La(NO_3)_3{\cdot}6H_2O$ (1/1) in 1.5 M DNO_3 in D_2O monitored over a week. The pictures showed the photographs of both ligand and the complexes at different time scales indicating no solid formed or precipitated out from the solutions.

From the NMR data given in Figure R1-R3, it could be concluded that both ligand and their corresponding La(III) and Lu(III) complexes displayed adequate stability in high acidity (1.5 M HNO_3) during a time period of a week.

To clarify the above discussions, we have added:

- Figure S20-S22 (Supporting Information);
- "*Also, **Phen-2DIBA** was stable in HNO_3 (1.5 M) during a period of a week as revealed by NMR data discussed in the NMR titration part.*" (Main Text, Page 5);
- "*At last, it's worthy to point out that the stabilities of the ligand, La(III) and Lu(III) related species were adequate stable in high acidity conditions (1.5 M HNO3) as indicated by NMR spectroscopy, no decomposition or precipitation were detected during a time period of a week (Figure S20-S22).*" (Main Text, Page 8, cross-referred to Figure S20-S22)

2. Figure S5 is not clear, and the current version cannot clearly display the structural information of hydrogen bonds. In addition, the authors should add a table indicating the bond length and angle information of hydrogen bonds.

Response 2: Figure S5 has been updated to clearly show the hydrogen bonds in **Phen-2DIBA**. Representative bonds as described in the main text were marked on the figure and the summary of bond lengths and bond angles were also added in Table S1. All added data was cross-referred in the main text (Page 5).

Added data:

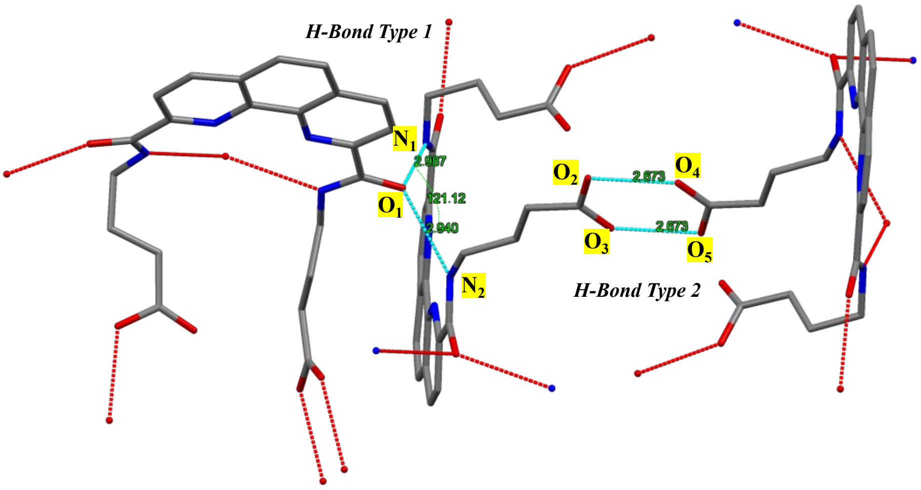

Figure R4. Molecular packing of **Phen-2DIBA** shows the hydrogen bonds formed between one oxygen atom and two N-H from another molecule. Two types of hydrogen bonds exist in the crystal, the second one forms between carboxylic groups. The distances shown on the figure give the bond lengths and angles between nonhydrogen atoms involved in the hydrogen bonds.

Table R1 Summary of hydrogen bonds in the crystal of **Phen-2DIBA**

Hydrogen Bond Type	Bonds	Distances	Angles
Type 1 (imide N-H to imide CO)	N_1-H-O_1	0.880; 2.259	140.01
	N_2-H-O_1	0.880; 2.261	133.91
Type 2 (carboxylic O-H to CO)	O_2-H-O_4	0.840; 1.837	172.52
	O_3-H-O_5	1.837; 0.840	172.52

3. In page 7, NaNO3 was used to control the ion strength in order to investigate the effect of nitrate ion on the extraction process. In page 9, Et4NNO3 was used to control the ion strength in order to get further information about the complexation behaviors of Phen-2DIBA with the trivalent metal ions in solution. Why did the authors use different nitrates to control the ion strength and whether different cations would affect the results?

Response 3: We thank the reviewer to bring this point into attention. In the current work, we did use $NaNO_3$ in the extraction while Et_4NNO_3 was used when discussed the complexation behaviors in absorption spectra titrations. Actually, both $NaNO_3$ and Et_4NNO_3 was used to control the ion strength in previous publications (**Revs**: *Chem. Rev.* **2013**, *113 (2)*, 1199-1236; *Inorg. Chem.* **2013**, *52 (7)*, 3414-3428; **Hydrophilic ligands**: *Solvent Extr. Ion Exch.* **2012**, *30 (5)*, 433-444; *Inorg. Chem.* **2021**, *60 (1)*, 357-365; **Lipophilic ligands**: *Inorg. Chem.* **2014**, *53 (3)*, 1712-1720; *Inorg. Chem.* **2021**, *60 (24)*, 19110-19119; just name a few). In some of the reported work, different nitrate salts were used in the discussions. For example, Shi and his coworker reported an acid-tolerance lipophilic phenanthroline derivatives and used both $NaNO_3$ and Et_4NNO_3 in their extraction and absorption titrations experiments (*Inorg. Chem.* **2021**, *60 (24)*, 19110-19119); another work from the same group, the author reported a hydrophilic sulfonated phenanthroline diimide ligand showed superior Eu(III)/Am(III) separation in 0.05 M HNO3, again, different nitrate salts of $NaNO_3$ and Et_4NNO_3 were used in their extraction and absorption titrations experiments (*Inorg. Chem.* **2021**, *60 (1)*, 357-365). From these results, we believe different nitrate salts would have little affect on both extraction and species distribution as in absorption titrations. Et_4NNO_3, itself, is quaternary ammonium salt and act as phase

transfer catalysis by its chemical nature, thus could potentially cause the third phase formation during extraction experiment. While, on the other side, Et_4NNO_3 shows good solubility in both aqueous and organic phase (inferior for $NaNO_3$), this will facilitate the absorption titration experiments especially for lipophilic ligands as the titrations are typically done in CH_3CN or methanol systems.

To specifically answer this question, we have done the absorption titration experiments under identical conditions except $NaNO_3$ was used instead of Et_4NNO_3, the results were given below (data updated in Figure S12 and Table S5, Supporting Information)

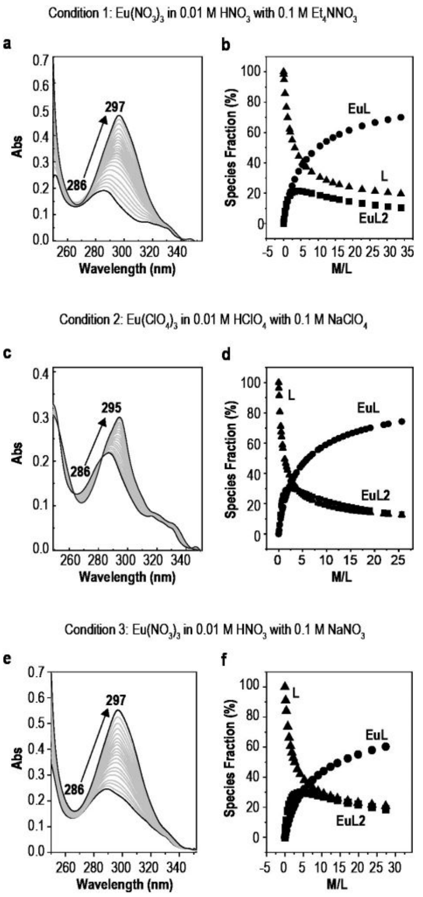

Figure R5. UV-vis absorption spectra titration of **Phen-2DIBA** with $Eu(NO_3)_3$ in 0.01 M HNO_3 (0.1 M NH_4NO_3) (a); $Eu(ClO_4)_3$ in 0.01 M $HClO_4$ (0.1 M $NaClO_4$) (c) and $Eu(NO_3)_3$ in 0.01 M HNO_3 (0.1 M $NaNO_3$) (e). The corresponding species distribution of Eu(III) with **Phen-2DIBA** derived from panel (a), (c) and (e) are given in (b), (d) and (f)

Table R2 Apparent stability constants for Eu(III) complexes with **Phen-2DIBA** obtained by UV-vis absorption spectra titrations in different ionic medium at 25 ^{o}C

Ionic medium	Ions	Reactions	Log β
0.01 M $HClO_4$ with 0.1 M $NaClO_4$	Eu^{3+}	$L + Eu^{3+} \rightleftharpoons EuL^{3+}$	4.36±0.03
		$2L + Eu^{3+} \rightleftharpoons EuL_2^{3+}$	10.33±0.52
0.01 M HNO_3 with 0.1 M $NaNO_3$		$L + Eu^{3+} \rightleftharpoons EuL^{3+}$	4.31±0.03
		$2L + Eu^{3+} \rightleftharpoons EuL_2^{3+}$	9.47±0.02
0.01 M HNO_3 with 0.1 M NH_4NO_3		$L + Eu^{3+} \rightleftharpoons EuL^{3+}$	4.36±0.07
		$2L + Eu^{3+} \rightleftharpoons EuL_2^{3+}$	9.49±0.19

From the above data, we could conclude that the similar results were observed for the two cations. At last, to avoid misleading the readers, we have changed the data in Figure 3 (main text, Page 9) to the data with $NaNO_3$ (to make it consistent with extraction experiment) and data with NH_4NO_3 and that in ClO_4^- were moved to Supporting Information (Figure S12 and Table S5). The updated data were also cross-referred in the main text by adding *"titration data in non-coordination solvent $HClO_4$ and NH_4NO_3, apparent stability constants for both titrations in HNO_3 and $HClO_4$ were given in Figure S12 and Table S5"* (Page 7)

4. Page 10, the understanding of 'red-shifted' and 'blue-shifted' is incorrected.

Response 4: We are sorry that we have not got the reviewer's point on this comment. On Page 10 of the original manuscript, the only places that red- and blue-shifted were mentioned were when we discussed the IR shifts: "*As given in Figure 4a and S20, after coordinating with Eu(III) ions, the C=O (imide) peaks at 1650 cm^{-1}* ***red-shifted*** *to 1636 cm^{-1} while the C=N peaks (phenanthroline) at 1549 cm^{-1}* ***blue-shifted*** *to 1570 cm^{-1}*". Note that in IR spectra, wavelengths were used as x-axis, so when the energy of a bond weakened, it's red-shifted given smaller wavenumbers. While, to avoid misleading the readers, we have changed all red-shifted and blue-shifted terminology into "shifted".

5. Page 10 and page 11, 'The 10-coordinated architectures were common for Lns(III)/Ans(III) complexes while, to the best of our knowledge, this was the first report on this kind of dimer-like two-metal-two-ligand coordination mode for Eu(III) complexes.' Binuclear rare earth complexes are very common. Phen-2DIBA-Eu2 is not the first Eu(III) complex with 'dimer-like two-metal-two-ligand coordination mode'. Several Eu(III) complexes (CCDC NO. 1059019, 1953922, 832850…) exhibit similar coordination mode.

Response 5: We totally agree with the reviewer on this point. The dimer-like Eu(III) complexes were reported in the literatures: except for the examples given by the reviewer (Eu dimer for sensing), other cases included Eu-dimer for construction of supramolecular architectures (*Chem. Rev.* **2022**, *122 (6)*, 6374-6458; *Angew. Chem. Int. Ed.* **2012**, *51 (45)*, 11302-11305; *J. Am. Chem. Soc.* **2004**, *126 (30),* 9413-9424; et al.); for LED and energy transferring (*Coord. Chem. Rev.* **2009**, *253 (21)*, 2627-2638; *Chem. Soc. Rev.* **2013**, *42 (8)*, 3278-3288; *Angew. Chem. Int. Ed.* **2014**, *53 (28)*, 7259-7263; *Inorg. Chem.* **2003**, *42 (22)*, 6977-6979; et al.).

Our emphasis here in the current manuscript, the dimer-like Eu-coordination mode was first-of-its-kind observed for Lns(III)/Ans(III) separation, and we did believe, this dimer-like structure contributed to the acid-resistance of the ligand. To be more specific, we changed the wording to "*while, to the best of our knowledge, this was the first report on this kind of dimer-like two-metal-two-ligand coordination mode for Eu(III) complexes observed for hydrophilic Lns(III)/Ans(III) separation phenanthroline diimide ligand (detailed crystal data for both* ***Phen-2DIBA*** *ligand and Eu(III) complexes were summarized in Table S8 and S9).*" (Page 10, main text)

6. PXRD of complex Phen-2DIBA-Eu2 should be provided to confirm purity. Cif and checkcif documents should also be provided.

Response 6: PXRD data for both ligand **Phen-2DIBA** and **Phen-2DIBA**/Eu(III) complexes were given as below:

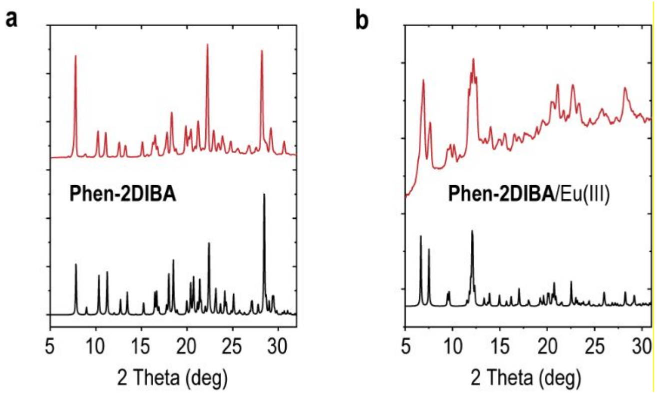

Figure R6. Comparison of PXRD data (red traces) and powder diffraction data derived from single X-ray data (black traces) of **Phen-2DIBA** (a) and **Phen-2DIBA**/Eu(III) (b).

The data was updated as Figure S28 (Supporting Information) and cross-referred in the main text as "*The ensemble level purities of both **Phen-2DIBA** ligand and **Phen-2DIBA**/Eu(III) complexes were examined by powder X-ray diffraction (PXRD), the results were given in Figure S28 and compared to the simulated results derived from single crystal X-ray diffraction data. The overall matches of the two data sets indicated the structural similarities of ensemble sample and the single X-ray diffraction results*." (Main text, Page 10)

7. The authors need to figure out the effect of different concentrations of salt agents on the masking effect.

Response 7: We actually have these data in the original version of the manuscript. In Figure 2 of the main text, panel a showed the distribution ratios and separation factors obtained in the extraction of Am(III) and Eu(III) by **TODGA** with **Phen-2DIBA** as a function of acid concentrations. We controlled the total concentration of nitrate anions to be 3 M, thus when the overall HNO_3 concentration increased from 1 M to 3 M, indicating the concentrations of $NaNO_3$ decreased from 2 M to 0 M.

To clearly show the effect of nitrate ion strength on the extraction, we fixed the HNO_3 to 1.5 M (highest *SF*) and examined the $NaNO_3$ concentrations on both the distribution and *SF* of Am(III)/Eu(III), the results were given below:

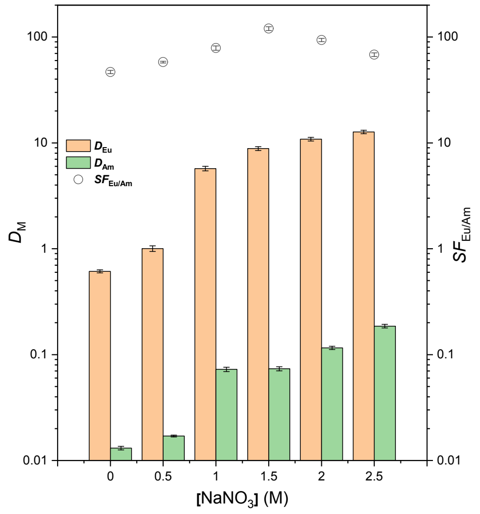

Figure R7. Extraction of Am(III) and Eu(III) by **TODGA** in the presence of **Phen-2DIBA** as a function of $NaNO_3$ concentrations. Organic phase: **TODGA** (0.05 M) in dodecane. Aqueous phase: 5 mM **Phen-2DIBA** in 1.5 M HNO_3. O/A=1; Vortex shaker (50 Hz) at 25±1°C.

This data was added as Figure S8 and cross-referred in the main text as "*The effect of nitrate ion strengths on the extraction performances of* ***Phen-2DIBA*** *were further demonstrated, the results were given in Figure S8.*" (Main text, Page 6)

8. The authors need to provide the relevant back-extraction experiments date. The authors need to clarify the effect of different masking agent concentrations on the back-extraction effect and make a relevant analysis of the back-extraction mechanism.

Response 8: To further demonstrate the Lns(III)/An(III) separation ability of the current reported ligands (in back-extraction experiments and the mechanism), the following experiments were conducted and the results were discussed in the Supporting Information (Note 1), also cross-referred in the main text as "*Additionally, the Eu(III)/Am(III) separation performances of* ***Phen-2DIBA*** *were further demonstrated in the stripping (back-extraction) experiment as described in i-SANEX process for direct comparison with the literature reported procedures (Supporting Information Note 1) and similar results as described above were observed.*" (Page 6)

Added experiments and discussions:

Supporting Information Note 1.
There were now mainly two different approaches using hydrophilic ligands for the separation of Lns(III) and Ans(III): the TALSPEAK process developed in the USA and hot tested in Sweden and i-SANEX process developed by French CEA and investigated in the European ACSEPT project. The former one used the combination of non-selective extraction agents such as ***TODGA*** *together with aqueous masking agents (water-soluble selective ligands), in which the masking agents selectively "held" certain metal ions to assist the extraction of* ***TODGA****, given the overall separation. Most of the literature hydrophilic ligands were reported with this process.*[3, 4, 5, 7, 8, 10] *In the second case, both Lns(III) and Ans(III) were coextracted by* ***TODGA****, then "stripping" agents were introduced to selectively back-extract certain metal ions, leaving the rest metal ions in the organic phase, given the overall separation.*[6, 11] *To further demonstrate the stripping performance of the current reported* ***Phen-2DIBA*** *ligand, the following stripping (back-extraction) experiments were conducted:*

Experiment 1: Effect of ***Phen-2DIBA*** *ligand concentrations on stripping of Am(III) from* ***TODGA****-based organic phases.*

0.05 M ***TODGA*** *in dodecane as organic phase (0.5 mL) were contacted with aqueous phase containing tracer amount of* ^{241}*Am(III) and* ^{152,154}*Eu(III) (0.5 mL, 3 M* HNO_3*) in closed glass tube. The mixture was vigorously shaken with vortex shaker for 30 minutes at 25 ± 1°C controlled with a water bath. After equilibrium, the two phases were separated by centrifugation at 3000 r/min for 2 minutes. The collected organic phases were contacted with aqueous phases containing different concentrations of stripping agents (1, 3, 5 mM of* ***Phen-2DIBA*** *in 1.5 M* HNO_3 *and 1.5 M* $NaNO_3$*). Same mixing and phase separation were repeated before aliquots were subsampled and analyzed, the relative concentrations of* ^{241}*Am and* ^{152,154}*Eu(III) in aqueous phases before and after extraction were measured using Liquid Scintillation Spectrometer (Quantulus 1220, PerkinElmer). The distribution ratio (D) was calculated by the ratio between the concentration (radioactivity counts per unit volume) in the organic phase and in the aqueous phase. The separation factors (SF) was determined by the ratio of distribution ratios of* ^{152,154}*Eu (III) to* ^{241}*Am(III).*

Table S3 Distribution ratios and separation factors for the stripping of Eu(III) and Am(III) from ***TODGA****-based organic phase into* ***Phen-2DIBA****-based aqueous phase*

Conc. of stripping agents	*Eu% in org*	*Am% in org*	$D_{Eu(III)}$	$D_{Am(III)}$	$SF_{Eu/Am}$
1 mM	*99.40*	*98.36*	*166.90*	*60.14*	*2.78*
3 mM	*98.66*	*67.40*	*73.36*	*2.07*	*35.44*
5 mM	*98.09*	*30.38*	*51.25*	*0.44*	*116.48*

Experiment 2: Effect of HNO_3 *concentrations on stripping of Eu(III) and Am(III) from* ***TODGA****-based organic phases.*

*As it's well known that the extraction abilities of **TODGA** increased with HNO_3 concentrations. Thus dilute HNO_3 solutions were used to back-extracted both Eu(III) and Am(III) from **TODGA**-based organic phases. After separation of the **TODGA** phases as described in the main text and extraction procedures in Page S3, the **TODGA** phases were contacted with different concentrations of dilute HNO_3 solutions, the mixing, phase separation and metal ions concentration quantifications were repeated as in experiment 1, the results were given in table S3, most of Eu(III) and Am(III) (over 99%) could be back-extracted into aqueous HNO_3 phases from **TODGA**-based organic phases.*

*Table S4 Distribution ratios for the stripping of Eu(III) and Am(III) from **TODGA**-based organic phase into dilute HNO_3*

Conc. of stripping HNO_3	*Eu% in org*	*Am% in org*	$D_{Eu(III)}$	$D_{Am(III)}$
0.1 M	*1.49*	*0.44*	*0.015*	*0.047*
0.05 M	*0.79*	*0.27*	*0.008*	*0.030*
0.01 M	*0.40*	*0.13*	*0.004*	*0.013*

9. The hydrophilic ligand only enhances the separation factor of TODGA appropriately, so please pay attention to the wording of the paper.

Response 9: We agree with the reviewer on this point that the current hydrophilic ligand increased the separation of non-selective **TODGA** by selective shielding certain metal ions (Am in the case), the overall increased $SF_{Eu(III)/Am(III)}$ was benefited from both ligands. Special attentions were paid to the related descriptions and marked in the main text and supporting information.

Ranking: Additional Questions:
Quality of experimental data, technical rigor: Top 5%
Significance to chemistry researchers in this and related fields: Top 5%
Broad interest to other researchers: High
Novelty: High
Is this research study suitable for media coverage or a First Reactions (a News & Views piece in the journal)?: Yes

Response: The author thanks the reviewer again for your precious time and efforts to review our work and for your positive comments. Also, we want to express our gratitude for your constructive feedbacks and suggestions. We have carefully addressed all the comments in a point-to-point manner and sincerely wish the current version is suitable for publication in ACS Central Science from your side.

Reviewer: 2
Recommendation: Reconsider after major revisions noted.

Comments: This work is interesting and can be potentially published in ACS Central Science after following suggested revisions.

Response: Thank you for reviewing our work and providing your insightful comments. We appreciate the time and effort you have dedicated to evaluating our paper. We have carefully considered your feedback and suggestions, and carefully address each of your comments as given below:

1. I can see the ligand design philosophy is similar to the previously proposed "hard/soft" combined strategy, where relatively soft N donor in phenanthroline and hard O donor in carboxylate are both utilized for Ln/Am coordination. The authors achieve record high separation factors of Eu/Am and Am/Cm in highly acidic condition by a masking effect in aqueous solution with such a hydrophilic ligand. A question needs to be considered is that does such a high separation capability originate majorly from the special ligand design or just from the unique masking method apart from the traditional solvent extraction. For instance, can author try a previously reported hydrophilic "hard/soft" ligand and try the masking separation to see the capability difference? This may provide a clue.

   Response 1: This is an interesting and valuable question. From our current data, we can hardly deny that the superior separation performance of the reported ligand in this work originated from hard/soft strategy. In fact, complexes crystallographic data as given in Table S7 clearly shown shorter Eu-O (around 2.5 Å) bonds than Eu-N (around 2.6 Å), indicating the contributions of "hard/soft" coordination of O and N sites. While we believed the superior separation performances was not solely contributed from hard/soft strategy for the following reasons:

   - Along with the preparation of reported ligand of **Phen-2DIBA**, two other ligands were also synthesized (Figure R1): **Refs 1** was prepared because it showed same number of carbons also we believe two hydroxyl groups would be comparable to one carboxylic group. While Refs 1 only displayed moderate discrimination ability towards Eu(III) and Am(III) with *SF* of around 10. In the other case, we prepared the mono-imide analogue of **Phen-2DIBA**, **Refs 2** was found to be even worse with almost no selectivity for Eu(III) and Am(III). From these two parallel experiments, we could conclude that except the binding sites (hard/soft O/N binding), the ending groups and the geometry of the ligand impact the overall performances to large extends.

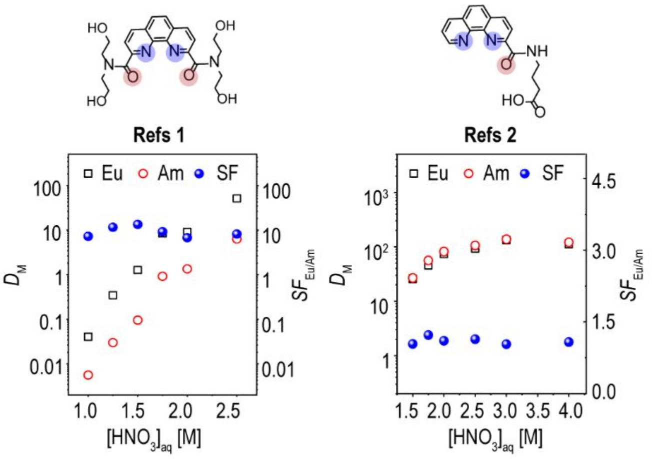

Figure R1. Eu(III)/Am(III) separation of two reference ligands under identical conditions as that of **Phen-2DIBA**.

   - We agree with the reviewer on the point of comparing with a reported hydrophilic "hard/soft" ligand to directly see the selectivity differences. To the best of our

knowledge, there were only two reported ligands bearing "hard/soft" binding sites. The **PhenDA-TriOH** reported by Scaravaggi showed limited solubility thus limited its applications (*J. Radioanal. Nucl. Chem.* **2015**, *303 (3)*, 1811-1820, reported $SF_{Eu/Am}$ of about 40). Also, **PhenDA-TriOH** was structurally similar to **Refs 1**. For **DS-Ph-DAPhen** reported by Ren (*Inorg. Chem.* **2021**, *60*, 357-365), as we do not have the ligand available in hand, we could not compare directly. While further examined the data in Ren's reports, **DS-Ph-DAPhen** displayed a $SF_{Eu/Am}$ of 170 when the acidity increased from 0.05 M HNO_3 to 0.3 M, thus we could expect that lower $SF_{Eu/Am}$ value with the acidity in the current work (1.5 M HNO_3).

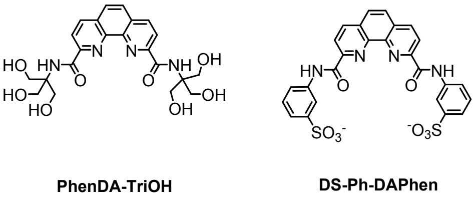

Scaravaggi, S. et al., *J. Radioanal. Nucl. Chem.* **2015**, *303*, 1811-1820. Ren, P. et al., *Inorg. Chem.* **2021**, *60*, 357-365.

Figure R2. Chemical structures of the two reported hydrophilic ligands with hard/soft binding sites.

- To investigate the masking methods on the superior performance of **Phen-2DIBA**, we further compared both the masking procedures and back-extraction procedures as these two procedures were the two different circumstances where hydrophilic ligands were tested in TALSPEAK process developed in the USA and *i*-SANEX process developed by French CEA individually. Detailed experiments were described in Supporting Information Note 1, the results were also cross-referred in the main text as "*Additionally, the Eu(III)/Am(III) separation performances of **Phen-2DIBA** were further demonstrated in the stripping (back-extraction) experiment as described in i-SANEX process for direct comparison with the literature reported procedures (Supporting Information Note 1) and similar results as described above were observed.*" (Page 6). The comparable *SF* data given from two different procedures could partially answer the reviewer's question on "*such a high separation capability originate majorly from the special ligand design or just from the unique masking method apart from the traditional solvent extraction*".

Added experiments and discussions:

"Supporting Information Note 1.

*There were now mainly two different approaches using hydrophilic ligands for the separation of Lns(III) and Ans(III): the TALSPEAK process developed in the USA and hot tested in Sweden and i-SANEX process developed by French CEA and investigated in the European ACSEPT project. The former one used the combination of non-selective extraction agents such as **TODGA** together with aqueous masking agents (water-soluble selective ligands), in which the masking agents selectively "held" certain metal ions to assist the extraction of **TODGA**, given the overall separation. Most of the literature hydrophilic ligands were reported with this process.*[3, 4, 5, 7, 8, 10] *In the second case, both Lns(III) and Ans(III) were coextracted by **TODGA**, then "stripping" agents were introduced to selectively back-extract certain metal ions, leaving the rest metal ions in the organic phase, given the overall separation.*[6, 11] *To further demonstrate the stripping performance of the current reported **Phen-2DIBA** ligand, the following stripping (back-extraction) experiments were conducted:*

*Experiment 1: Effect of **Phen-2DIBA** ligand concentrations on stripping of Am(III) from **TODGA**-based organic phases.*

*0.05 M **TODGA** in dodecane as organic phase (0.5 mL) were contacted with aqueous phase containing tracer amount of ^{241}Am(III) and ^{152,154}Eu(III) (0.5 mL, 3 M HNO_3) in*

*closed glass tube. The mixture was vigorously shaken with vortex shaker for 30 minutes at 25 ± 1°C controlled with a water bath. After equilibrium, the two phases were separated by centrifugation at 3000 r/min for 2 minutes. The collected organic phases were contacted with aqueous phases containing different concentrations of stripping agents (1, 3, 5 mM of **Phen-2DIBA** in 1.5 M HNO_3 and 1.5 M $NaNO_3$). Same mixing and phase separation were repeated before aliquots were subsampled and analyzed, the relative concentrations of ^{241}Am and $^{152,154}Eu(III)$ in aqueous phases before and after extraction were measured using Liquid Scintillation Spectrometer (Quantulus 1220, PerkinElmer). The distribution ratio (D) was calculated by the ratio between the concentration (radioactivity counts per unit volume) in the organic phase and in the aqueous phase. The separation factors (SF) was determined by the ratio of distribution ratios of $^{152,154}Eu$ (III) to $^{241}Am(III)$.*

*Table S3 Distribution ratios and separation factors for the stripping of Eu(III) and Am(III) from **TODGA**-based organic phase into **Phen-2DIBA**-based aqueous phase*

Conc. of stripping agents	Eu% in org	Am% in org	$D_{Eu(III)}$	$D_{Am(III)}$	$SF_{Eu/Am}$
1 mM	99.40	98.36	166.90	60.14	2.78
3 mM	98.66	67.40	73.36	2.07	35.44
5 mM	98.09	30.38	51.25	0.44	116.48

*Experiment 2: Effect of HNO_3 concentrations on stripping of Eu(III) and Am(III) from **TODGA**-based organic phases.*

*As it's well known that the extraction abilities of **TODGA** increased with HNO_3 concentrations. Thus dilute HNO_3 solutions were used to back-extracted both Eu(III) and Am(III) from **TODGA**-based organic phases. After separation of the **TODGA** phases as described in the main text and extraction procedures in Page S3, the **TODGA** phases were contacted with different concentrations of dilute HNO_3 solutions, the mixing, phase separation and metal ions concentration quantifications were repeated as in experiment 1, the results were given in table S3, most of Eu(III) and Am(III) (over 99%) could be back-extracted into aqueous HNO_3 phases from **TODGA**-based organic phases.*

*Table S4 Distribution ratios for the stripping of Eu(III) and Am(III) from **TODGA**-based organic phase into dilute HNO_3*

Conc. of stripping HNO_3	Eu% in org	Am% in org	$D_{Eu(III)}$	$D_{Am(III)}$
0.1 M	1.49	0.44	0.015	0.047
0.05 M	0.79	0.27	0.008	0.030
0.01 M	0.40	0.13	0.004	0.013

*Experiment 3: Comparison of extraction performances of **PyTri** and **Phen-2DIBA** under high acidity of 1.5 M HNO_3*

***PyTri** ligand represented one of the most important landmark ligands for hydrophilic Lns(III)/Ans(III) separation.[6] In this experiment, we compared the separation of Eu(III) and Am(III) of the two ligands side by side. Under the same condition as described in the main text, PyTri gave $D_{Eu(III)}$ and $D_{Am(III)}$ of 1.539 and 0.917 with the corresponding $SF_{Eu/Am}$ of 1.68. (Conditions: Organic phase: **TODGA** (0.05 M) in dodecane. Aqueous phase: 5 mM **Phen-2DIBA** or **PyTri** in 1.5 M HNO_3 with 1.5 M $NaNO_3$. O/A=1; Vortex shaker (50 Hz) for 30 minutes at 25±1°C)"*

2. Another critical data authors may seriously consider to obtain (if authors have such a capability ideally, or at least DFT calculation with a model assuming Am/Eu structures are isotypic) is to grow the single crystal of Am complexes with the new ligand, determine the crystal structure, and compare the Am-N, Am-O distances to Eu-N, Eu-O (maybe Nd-N, Nd-O is better because Am3+ has nearly identical ionic radius with Nd3+, not Eu3+), and then compare these data with other well studied ligand systems.

This would tell much better whether the ligand design amplifies the 4f/5f bonding difference and the advantages.

Response 2: We totally agree with the reviewer's suggestion on this point while cultivating single crystals of radioactive Am complexes were both technically and practically hard to accomplish. Nevertheless, we have indeed tried to grow Am/**Phen-2DIBA** crystal with ~ 0.5 mg of Am-241, but no measurable crystal was obtained. We have done the DFT calculations of both Eu, Am and Nd with newly reported **Phen-2DIBA** ligand, the results were added as Supporting Information Note 3 and cross-referred in the main text as "*To better under the binding differences of **Phen-2DIBA** ligand with Eu(III) and Am(III), we calculated the bond length on the optimized complexes geometries with a simplified 1:1 architecture of [$M(NO_3)_3L$]. the results were discussed in Supporting Information Note 3 (Table S10). The relative longer Eu-N and shorted Eu-O bonds in comparison with Am-N and Am-O agreed well with the reported data for other hydrophilic ligands,[24] indicating the softer nature of Ans(III).*"

While from solely the DFT data, we can hardly say that the current ligand design amplified the 4f/5f bonding differences and thus gave the superior performances. As from our discussions in Supporting Information Note 3 and bond lengths data in Table S10, the differences of average Am-N and Eu-N in our case was 0.04 (longer for Eu-N), and Eu-O and Am-O was 0.03 (longer for Am-O). While similar data in closely related reported systems of **DS-Ph-DAPhen** were 0.03 and 0.04 (*Inorg. Chem.* **2021**, *60*, 357-365). The author believed that the calculated data was inadequate to give the conclusion that our current ligand design amplified the 4f/5f differences. While, we did think the first explanation given in discussion part of the main text could shed some light on the superior performance of **Phen-2DIBA**: **Phen-2DIBA** chose a dimer-like architecture to fulfill the entropy requirement (three ligands other than four around metal centers) and binding stabilities (more O binds to metal centers) simultaneously, leading to the superior selectivity.

3. There are several important progresses in Am/Ln separation that are missed in the reference list.

Response 3: We have further added the following progresses in the introduction parts: oxidation states control for Lns/Ans separation - *Nat. Commun.* **2023**, *14* (1), 261; theoretical and experimental progresses for Lns/Ans separation lipophilic ligands - *Inorg. Chem.* **2023**, *62* (6), 2705-2714; *Chem. Commun.* **2022**, *58* (76), 10667-10670; *J. Environ. Chem. Eng.* **2022**, *10* (5), 108401; theoretical and experimental progresses for Lns/Ans separation hydrophilic ligands - *Sep. Purif. Technol.* **2023**, *319*, 124030; *Inorg. Chem.* **2023**, *62* (11), 4581-4589; *J. Environ. Chem. Eng.* **2023**, *11* (2), 109536.

Ranking: Additional Questions:
Quality of experimental data, technical rigor: Top 5%
Significance to chemistry researchers in this and related fields: Top 5%
Broad interest to other researchers: High
Novelty: High
Is this research study suitable for media coverage or a First Reactions (a News & Views piece in the journal)?: Yes

Response: The author would thank the reviewer again for the valuable insights and contributes to the improvement of current manuscript. We could be thrilled to know that the reviewer could agree with our responses to your concerns and wish you would now agree the acceptance of this work to be published in ACS Central Science.
